# Cereal Fibers and Satiety: A Systematic Review

**DOI:** 10.1093/nutrit/nuaf083

**Published:** 2025-07-11

**Authors:** Alicia Machalias, Jessica J A Ferguson, Trish Guy, Eleanor J Beck

**Affiliations:** School of Medical, Indigenous and Health Sciences, University of Wollongong, Wollongong, New South Wales 2522, Australia; Sanitarium The Health Food Company, Berkeley Vale, New South Wales 2261, Australia; Sanitarium The Health Food Company, Berkeley Vale, New South Wales 2261, Australia; School of Medical, Indigenous and Health Sciences, University of Wollongong, Wollongong, New South Wales 2522, Australia; School of Health Sciences, University of New South Wales, Sydney 2052, Australia

**Keywords:** cereals, cereal fibers, grains, whole grain, satiation, satiety, appetite, hunger

## Abstract

**Context:**

Intake of cereal fiber has been linked to favorable health outcomes, such as lower body weight. Changes in perceived appetite sensations are a possible mechanism. Evidence of varied effects of different cereal fibers on satiety is conflicting.

**Objective:**

Considering satiety as a potential mechanism to reduce dietary intake, this study aimed to systematically review the effect of higher cereal fiber intake compared with a lower-fiber control on appetite sensations and ad libitum energy intake, using visual analog scales and subsequent meal intake data.

**Data Sources:**

Literature pertaining to the effects of cereal fibers on satiety outcomes was gathered through Medline, Scopus, CINAHL Plus, and Web of Science.

**Data Extraction:**

Randomized crossover intervention studies in healthy humans assessing the effects of cereal fibers on subjective measures of appetite for a minimum of 2 hours, with ≥3-day washout periods, and that detailed the type and amount of fiber delivered for each intervention were eligible. Quality was assessed using the Academy of Nutrition and Dietetics Quality Criteria Checklist and Health Canada Consistency Tool.

**Data Analysis:**

Evidence from 48 studies indicated that cereal fiber intake was associated with favorable effects on satiety and other measures of appetite but limited effects on ad libitum energy intake. Higher cereal fiber intake from rye and oat sources showed superior effects on appetite compared with a lower-fiber control. Wheat and barley fibers as well as functional fibers, resistant starch, and soluble corn fiber, showed a weak effect on appetite sensations and ad libitum energy intake.

**Conclusion:**

Overall, a higher intake of cereal fiber compared with a low-fiber control indicates positive effects on satiety measures. Further research is required to assess the influence of physicochemical properties of different cereal fiber types as well as effects of age, gender, and disease state on expression of satiety signals.

**Systematic Review Registration:**

PROSPERO registration no. CRD42023395182.

## INTRODUCTION

In 2017–2018, more than two-thirds (67%) of Australians aged 18 years and older were considered overweight or obese.^1^ This may be attributed to a range of multifaceted etiological drivers, most notably the growing emergence of obesogenic environments through which energy-dense foods are increasingly available, accessible, and affordable.^2^ In contrast, foods that are lower-calorie yet nutrient-dense and particularly rich in dietary fiber have been linked to lower body mass index (BMI), body fat percentage, and waist circumference.^3–6^

Cereal grains are defined as the edible seeds from the plants of the Poaceae (grass family) and comprise bran, germ, and endosperm fractions.^7^ Commonly consumed grains include rice, wheat, maize, oats, rye, barley, millet, and sorghum. Cereals are considered a staple food across both developed and developing countries, providing carbohydrates, protein, energy, and fiber in addition to a variety of micronutrients such as B vitamins, vitamin E, zinc, and magnesium as well as bioactive compounds including phytosterols and polyphenols.^8^^,^^9^ In particular, whole-grain foods, high in cereal fiber, are consistently linked to lowered risk of chronic disease.^10^^,^^11^ Individuals with higher cereal fiber intakes tend to have lower body weight,^12^ and increased satiety via cereal fiber ingestion may be a potential mechanism for reduced body-weight gain.^13^^,^^14^ Satiety is referred to as the sensation of fullness that lingers post–meal ingestion, delaying prospective hunger and consumption. That is, the mechanism through which whole grains and cereal fiber impact body weight over a long-term period may, in part, be through appetite suppression and a resultant reduction in energy intake.^15^^,^^16^

Many high-fiber-grain foods have the propensity to influence gastrointestinal transit, hormone secretions, and glucose metabolism, all of which have the capacity to impact appetite.^17^^,^^18^ Fiber increases mastication, which hinders intake through the promotion of salivary and gastric acid secretion, resulting in abdominal distention and increased satiety.^19^^,^^20^ The viscosity and bulking properties of dietary fiber can also greatly decrease the rate of ingestion of the food.^21^ Increased digesta viscosity has been found to slow small intestine transit time as well as its nutrient absorptive capacity, thereby limiting postprandial blood glucose and lipid concentrations,^19^^,^^21^ mediating appetite hormone release, and increasing satiety.^22^^,^^23^ Comparatively, insoluble fiber provides fecal bulk and decreased transit time^24^; however, its role in the regulation of appetite necessitates further research.

Alterations to the food structure of cereal grains through processing techniques such as milling have the propensity to impact satiety.^25^ Less refined (intact) cereals are linked to a longer sensation of satiety, which may be indicative of the role that particle size plays in metabolic pathways.^26^ The molecular weight (MW) of fibers is guided by the degree of processing, altering viscosity, which also has been shown to affect appetite regulation.^27^

Despite these findings, evidence from systematic reviews examining the relationship between fiber and satiety has varied.^28^^,^^29^ Clark and Slavin^28^ reviewed the acute effects (<24 hours) of various cereal fiber types on satiety response and food intake. It was reported that most of the cereal fiber interventions were ineffective and neither the fiber dose nor type influenced the participants’ satiety response or energy intake. Poutanen et al^30^ found that dietary fiber that increased in viscosity upon digestion had the most beneficial effects on appetite sensations, with fermentable (more soluble) dietary fiber proving least efficacious. Therefore, the physicochemical properties of a fiber may be more important than the specific fiber type.

Subjective feelings of appetite encompass hunger, fullness, and satiety, as well as the urge to eat and an individual’s willingness for prospective food consumption.^31^ A validated measure to assess subjective sensations of appetite are visual analog scales (VASs), which comprise a spectrum of words describing an extreme at either end of the line. The VASs for the purpose of assessing appetite have proven to be reproducible and reliable in validation studies.^31–34^ Ultimately, optimizing satiety is an important strategy that may facilitate reduced energy intake. Therefore, examination of satiety measures and ad libitum dietary intake at subsequent meals may serve as a relatively noninvasive, cost-effective method to further elucidate appetite-related mechanisms that may underpin weight management.^35^^,^^36^ While longer-term studies may describe outcomes, understanding physiological effects of foods acutely provides an understanding of the potential mechanisms by which outcomes are achieved. This may further inform dietary guidelines or food product development.

Cereals and grains are the primary source of energy and carbohydrates worldwide.^37^ Given high-fiber cereal foods link to lowered body weight, this review aimed to systematically assess the effect of cereal fiber intake on appetite sensations in humans using a subjective measure of appetite (VAS), with ad libitum energy intake as a secondary outcome. Additional outcomes pertaining to the effects of cereal fiber types and sources of cereal fiber, including added fibers (isolated fibers added to foods—hereafter, extrinsic fibers) and cereal fiber as part of whole-grain foods, were also explored.

## METHODS

### Search Strategy

The protocol was registered in the PROSPERO International Prospective Register of Systematic Reviews (CRD42023395182) and followed the Preferred Reporting Items for Systematic Reviews and Meta-Analyses (PRISMA) guidelines and checklist.^38^ Literature pertaining to the effects of cereal fibers on satiety outcomes was gathered through searches in Medline, Scopus, CINAHL Plus, and Web of Science. A preliminary investigation of the literature was conducted in Medline to determine scope and relevant key words. The final search strategy (“edible grain” OR cereals OR poaceae OR “whole grain” OR wholegrain OR “dietary fiber”) AND (appetite OR satiety OR satiation OR “satiety response”) was undertaken in February 2023 with no date restrictions. This was repeated in March 2024 to update the search; however, none of the recovered studies were deemed to meet the inclusion criteria. Inclusion criteria included full-text publications in humans and adults and in English language. The search terms included cereals, poaceae, edible gain, wholegrain, dietary fiber, appetite, satiety, satiation, and satiety response. The full search strategy used for each database is available in Table S1. Data obtained from the searches conducted on each database were imported and managed in EndNote X20.4 (2022; Clarivate Analytics, Philadelphia, PA) by 1 assessor (A.M.). Covidence (Covidence Systematic Review Software, 2019; Veritas Health Innovation, Melbourne, Australia) was used for removal of duplicates and all phases of article screening.

### Study Eligibility, Selection Process, and Quality Assessment

Prespecified Population, Intervention, Comparator, Outcomes, and Study Design (PICOS) criteria were used to select studies for inclusion (Table 1). Screening of title and abstract, followed by the full text was undertaken by A.M., and then in duplicate by E.J.B. and J.J.A.F. using the PRISMA guidelines.^38^ Inclusion and exclusion criteria considered VAS measurements, duration of measurement, and the ability to determine fiber dose and relevant control (Table 2). Any differences in opinion between assessors were resolved through discussion with the third reviewer, who had not initially screened the article, until consensus had been reached. Where additional information was required for inclusion, authors were contacted up to 2 times, with articles excluded when there was no response.

**Table 1.** PICO Elements Used to Define the Research Question: In Generally Healthy Individuals (P), Does the Intake of Cereal Fibers or Cereals (I) Compared With a Lower Intake of Cereal Fibers or Cereals (C) Increase Satiety (O)?

PICO components	Key words
P (Population or Patient)	Healthy adults, healthy children, overweight/obese adults, adults with type 2 diabetes (non-insulin dependent)
I (Intervention	Cereal fibers or cereals
C (Comparison	Lower intake of cereal fibers or cereals
O (Outcome)	Increased satiety

**Table 2.** Criteria to Determine Inclusion or Exclusion of Potentially Eligible Studies

Inclusion criteria	Exclusion criteria
Randomized crossover trials	Studies other than randomized crossover trials, ie, non–systematic review articles, commentary, editorials (secondary information)
Population group: healthy adults, healthy children, overweight/obese adults and adults with type 2 diabetes (non-insulin dependent)	Individuals with insulin-dependent diabetes or medical conditions other than those listed in inclusions or where there were any significant uncontrolled changes during the trial known to impact appetite (diagnosis of illness, menstruation in women, pregnancy, smoking)
Monitoring of VAS ≥2 h and up until 24 h or meal intake data for the same period	Measurement period <2 h
Grains and cereals (as per definition) were tested (other food components in the same study were acceptable provided that the effects of the cereals/cereal fiber could be separated)	The fiber was not cereal fiber, cereal fiber was not the only fiber added to the intervention tests (ie, psyllium and fiber supplements or the intervention contained a mixed-source dietary fiber whereby the cereal fiber effect alone could not be distinguished)
The amount of grains or cereal fiber consumed was clearly declared/measured	Fiber dose was not clearly stated
An appropriate control group test was performed; the control food was similar to the intervention, was delivered through the same mode (ie, via a capsule, whole-food, etc), of similar nutritional composition, or was a low-fiber version of the same food	Not sufficiently or appropriately controlled: delivery modes significantly differed, ie, powder capsule compared with liquid fiber drink, no control group, or unsuitable control test meal or tested only against glucose
Measurements of VAS were included; studies with biochemical markers (ie, appetite hormones) were included only where VAS and/or dietary intake data were available	Outcome measurement other than VAS measuring appetite, biomarkers (appetite hormones), and energy intake
Appropriate washout period between tests (ie, ≥3 d)	Insufficient washout period between interventions (ie, <3 d)
Studies that included a magnitude of the effect or provided sufficient data to be able to calculate magnitude of effect	Inadequate information provided to determine the magnitude of the effect: no measure/estimate of cereal fiber intake or limited information pertaining to outcome measures
Adequate fasting period (ie, ≥10 h prior to intervention)	Fasting period <10 h prior to intervention
—	Study examined an entirely irrelevant food/diet and health relationship in relation to the one under consideration
—	Study was duplicated, ie, study population was the same as one of the studies to be used as evidence or was a pilot study for a larger study to be included

Abbreviation: VAS, visual analog scale.

Cereal grains, as well as pseudo-cereals (ie, non-poaceae plants such as quinoa with similar properties and uses)^39^ could be consumed in a variety of forms, including as a whole food, processed, added as an ingredient to a test product, or isolated and administered in supplement form. Intervention arms that differed significantly due to mode of delivery and those containing mixed sources of dietary fibers and differences in food matrices and nutritional composition along with inappropriate controls (eg, glucose syrup) were not extracted or included in the analysis.

The assessment of methodological quality was completed by A.M. using the Quality Criteria Checklist (QCC) tool for Primary Research in the Academy of Nutrition and Dietetics’ Evidence Analysis Manual.^40^ The QCC enabled comprehensive appraisal of the validity and significance of selected publications as it contains 10 structured validity questions and additional sub-questions specific to different types of research designs. The risk of bias and scientific quality were assessed via the following validity criteria contained in the QCC: research question; selection of participants; comparability of study groups; withdrawal handling; blinding methods (if any); description of intervention, procedures, and intervening factors; definition of outcomes, including validity and reliability of measurements; statistical methodology; conclusions; and biases and limitations considering any funding or sponsorships. An overall systematic and objective rating (ie, positive, negative, or neutral) was assigned to each publication. Duplicate assessment of quality was conducted for a random 20% of the studies by J.J.A.F., T.G., and E.J.B. to ensure integrity of the data and consistency of quality assessment.

### Data Extraction

Data extraction was conducted by A.M. and data tabulated and managed using Excel version 16.70 (Microsoft Excel Spreadsheet Software 2023; Microsoft Corporation, Redmond, WA) with the following key components extracted: author, publication date, country of origin, study aim/objective(s), study design, presence of blinding, total sample size (including proportion of males and females), mean age, BMI, population condition, study inclusion/exclusion criteria, study setting, fasting period, study duration, washout period, total number of test days, duration of VAS measurement, number of dropouts per allocation group, details of control and each intervention, number included in analysis of results, primary and secondary outcome(s), details of ad libitum food intake, confounders, and any adverse effects. Means or medians and SDs or SEMs as reported by the authors were also collated.

### Qualitative Data Synthesis

The Health Canada Consistency Tool (HCCT) was used to grade the overall consistency of the studies for each outcome.^41^ The HCCT uses the outcomes of all studies to produce a grading based on the consistency of results and direction of the effect, with a high consistency rating of 75% or greater, moderate of 60%–74%, and low of less than 60%.^41^ Individual intervention arms were used to generate the consistency ratings rather than the total number of studies, to ensure variation in outcomes between each treatment arm was considered. The HCCT was used to compare the proportion of intervention arms with a favorable direction of effect (whether statistically significant or non–statistically significant) by the total number of intervention arms. This was undertaken for higher-quality studies only. The strength of evidence for each outcome was based on the proportion of intervention arms showing statistical significance (defined as *P* < .05) among all included studies for that outcome, according to the Health Canada Guidance Document for preparing a submission for food health claims.^41^ This was again only undertaken for higher-quality studies. A favorable direction of effect for each appetite sensation was considered as decreased hunger, desire to eat and prospective food consumption, and increased fullness and satiety.

A generalized analysis of all intervention arms and associated effects on appetite sensations and ad libitum energy intake was completed using the HCCT. Subgroup analysis by cereal fiber types, including grain type, extrinsic fibers, and cereal fiber as part of whole-grain foods, explored potential differences in associated effects.

## RESULTS

### Overview of Publications

**Figure 1.**
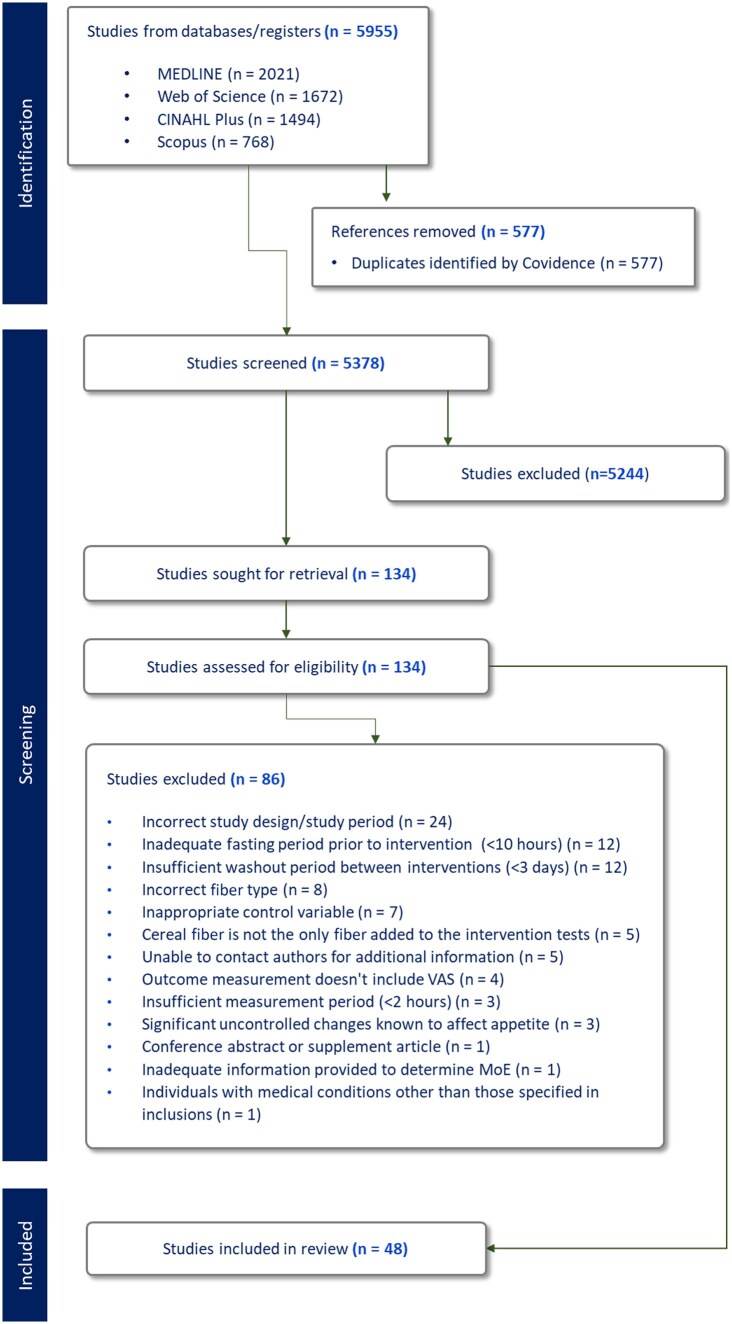
PRISMA (Preferred Reporting Items for Systematic Reviews and Meta-Analyses) Flow Diagram Outlining the Process of Study Selection. Abbreviations: MoE, measure of effect; VAS, visual analog scale

The search yielded a total of 5378 articles after the exclusion of duplicates. After completion of screening and full-text review, 48 randomized crossover studies were deemed appropriate for inclusion (Figure 1). Key reasons for exclusion were incorrect study design or study period, inadequate fasting period prior to intervention (<10 hours), and insufficient washout period between interventions (<3 days). Of the 48 included studies, a total of 4 were missing information^42–45^ that was unable to be clarified after attempts to contact the original authors. Fasting period undertaken by participants was not specified in 3 articles,^46–48^ and 1 article omitted details relating to the duration of washout between interventions.^49^ Studies were also excluded if the fiber dose/nutritional composition of test products was unable to be determined. A total of 98 intervention arms were derived from 48 studies and were analyzed and assessed according to appetite sensations and related outcomes including hunger, desire to eat, fullness, satiety, prospective food consumption, and ad libitum energy intake (Table 3).

**Table 3.** Summary Table of Participant Characteristics, Individual Intervention Arms, and Corresponding Portion Size, Energy Content, and Total Fiber Dose and Associated Appetite and Ad Libitum Energy Intake Outcomes for Included Studies

			Participant characteristics					Intervention characteristics				Outcome									
Study (year) (quality rating)	Blinding	Study objectives	**Sample size** ^a^ **(drop-out no.)**	Mean age (y)	**Mean BMI (kg/m** ^2^ **)**	Confounders measured	Measurement tool (duration)	Intervention and control food(s)	Serving size (g)	kJ	**Fiber dose (g)** ^b^	Hunger	Fullness	Satiety			DTE		PFC	Ad libitum EI	Adverse effects
Alyami et al (2019A) (+)^50^	N	Investigate the effect of a PMP compared with a well-known SOP on glycemic, gastrointestinal, hormonal, and appetite responses	26	28.5	23.4	1-wk washout, ≥12-h fast, habitual intake between tests	VAS (2.25 h)	Pearl millet porridge	NR	920	IF: 6.4 SF: 3.0 βG: 1.6	↓	↑	↑			↓		↓	━	N
								Scottish oats porridge (control)^c^	NR	920	IF: 3.1 SF: 4.9 βG: 2.9										
Alyami et al (2019B) (+)^51^	N	Collect initial pilot data on the physiological and gastrointestinal responses to breakfast porridges made with 2 millet varieties and oats and rye grains	16 (-1)	20.9	22.1	1-wk washout, ≥10.- h fast, habitual intake between tests, new VAS sheet provided at each measurement point	VAS (2 h)	Finger millet porridge	432.0	920	TF: 13.8	↓	↑	↑			↓		↓	NR	N
								Scottish oats porridge	400.0	920	TF: 8.0	↓	↑	↑			↓		↓	NR	
								Pearl millet porridge	311.0	920	TF: 6.8	⇊	↑	↑			↓		↓	NR	
								Rye porridge (control)^c^	297.0	920	TF: 6.5										
Aoe et al (2014) (+)^52^	N	Investigate the effect of cooked white rice containing high BAR on appetite and EI	22 (-1)	41.9	23.3	1-wk washout, ≥12-h fast	VAS (4 h)	β-glucan enriched barley	150.0	879	TF: 5.3 βG: 2.9	⇊	⇈	↑					⇊	⇊	N
								White rice (Control)^c^	147.0	879	TF: 0.0										
Barone Lumaga et al (2012) (+)^53^	Y	Investigate the satiating effect of 3 beverages containing different sugar and dietary fiber composition and monitor subsequent appetite sensations, blood profile secretion of 6 GI hormones and EI	14	27.8	20.2	1-wk washout, ≥10.5-h fast, menstrual cycle phase, presence of physical/psychological discomfort	VAS (3 h)	β-Glucan enriched beverage	250 (mL)	617	βG: 3.0	⇊	⇈	⇈						⇊	N
								Tropical fruit flavored beverage (control)	250 (mL)	624	βG: 0.0										
Beck et al (2009) (+)^54^	N	Investigate the effect of β-glucan in extruded breakfast cereals on acute satiety, the dose responsiveness of such effects, and whether differing processing methods of β-glucan modulates acute satiety responses	14 (-3)	38.7	29.6	3-d washout, ≥10-h fast, menstrual cycle phase, 24-h dietary recall prior to test	VAS (4 h)	β-Glucan cereal (low)	45.0	1098	TF: 3.7 βG: 2.16	↓	⇈	⇈				↓		NR	N
								β-Glucan cereal (mid)	45.0	1106	TF: 6.7 βG: 3.82	↓	⇈	⇈				↓		NR	
								β-Glucan cereal (high)	45.0	1115	TF: 9.7 βG: 5.45	↓	⇈	⇈				↓		X	
								β-Glucan cereal (extracted)	45.0	1157	TF: 7.8 βG: 5.65	↓	⇈	⇈				↓		⇊	
								Corn flakes cereal (control)	39.0	1080	TF: 1.2 βG: 0.0										
Belobrajdic et al (2019) (+)^55^	Y	Determine the effects of bread made from HAW and enriched in RS on postprandial glycemia compared with bread made from conventional LAW	19 (-1)	30.0	23.0	1-wk washout, 12-h fast, 24-h dietary recall prior to test	VAS (3 h)	High-amylose wheat whole-meal bread	121.0	922	TF: 10.4			⇈		⇊					N
								High-amylose wheat refined bread	121.0	951	TF: 5.5			⇈		⇊					
								Low-amylose wheat whole-meal bread	121.0	984	TF: 8.2			⇈		⇊					
								Low-amylose wheat refined bread (control)	121.0	1045	TF: 3.3			⇈		⇊					
Berti et al (2015) (ø)^56^	Y	Measure the effect of isoenergetic breakfasts varying in cereal-based food types on satiety-related sensations in adults	9	25.0	20.5	1-wk washout, 10.5-h fast	VAS (3.5 h)	Whole-wheat flakes	60.0	1339	TF: 9.0			⇈		⇊					N
								Corn flakes cereal (control)^c^	55.0	1359	TF: 1.4										
Bodinham et al (2010) (+)^57^	Y	Investigate the acute effects of consuming 48 g of RS type 2 on EI, subjective appetite measures, postprandial glucose and insulin compared with a carbohydrate matched placebo supplement	20	25.8	23.2	1-wk washout, 12-h fast	VAS (3 h)	Resistant starch mousse	67.2	1595	TF: 24.0	━	━			━		━		⇊	N
								Mousse (control)	67.2	1595	TF: 0.5										
Breen et al (2013) (+)^58^	N	Examine the effect of commonly consumed breads among adults with T2DM on postprandial blood glucose, insulin, and appetite responses	11 (-1)	53.9	35.1	1-wk washout, 12-h fast	VAS (4.5 h)	Whole-meal soda bread	131	1233	TF: 7.4	━	━	━				━			Y^d^
								Whole-grain bread	140	1364	TF: 7.5	━	━	━				━			
								Pumpernickel rye bread	196	1492	TF: 19.2	━	⇈	⇈				━			
								Wheatenwhite bread (control)^c^	124	1145	TF: 3.4										
Costabile et al (2018) (+)^59^	N	Identify acute strategies for the regulation of appetite and improvement of glucose control through the use of different pasta meals	14	30.0	22.0	1-wk washout, 12-h fast, 24-h dietary recall prior to test	VAS (4 h)	Whole-meal pasta	117	1431	TF: 11.0	⇊		⇈		⇊				↓	N
								Refined-wheat pasta (control)	100	1322	TF: 3.0										
Emilien et al (2017) (+)^60^	Y	Investigate the effect of replacing 40% of all-purpose flour with an equal weight of RS wheat flour in muffins on EI, subjective appetite, biomarkers of appetite, and glycemic response	31 (-4)	23.0	23.5	1-wk washout, 10-h fast	VAS (4 h)	Resistant wheat starch muffin	NR	2900	TF: 26.0	━	━			━		━		⇊	N
								Standard wheat flour muffin (control)	NR	3251	TF: 2.0										
Flint et al (2006) (+)^61^	N	Test whether postprandial appetite responses and subsequent EI are influenced by postprandial glycemic and insulinemic responses post-consumption of a variety of breakfast meals	28	24.8	22.5	≥1-wk washout, 11-h fast	VAS (3 h)	German bread^e^	216	2843	TF: 5.0	⇊	NR	NR				⇊		━	N
								Finnish bread	198	2882	TF: 10.0	⇊	NR	NR				⇊		━	
								White wheat bread (control)	194	2990	TF: 5.0										
Forsberg et al (2014) (+)^62^	Y	Investigate the acute effects on satiety, hunger, and DTE as well as EI after a regular breakfast with isoenergetic rye crisp bread (RB) or refined wheat bread (WB)	Study 1: 21 Study 2: 20	39.0	23.0	6-d washout, 12–13-h fast	VAS (4 h)	Whole-grain rye crisp bread	80.0	1188	TF: 13.0	⇊		⇈		⇊				━	N
								Refined-wheat bread (control)	108.0	1180	TF: 3.8										
								Whole-grain rye crisp bread	64.0	953	TF: 10.0	⇊		⇈		⇊				⇊	N
								Refined-wheat bread (control)	86.0	936	TF: 2.6										
Freeland et al (2009) (+)^63^	N	Assess the effect of equal portions of insoluble fiber (wheat bran) and glycemic carbohydrate (glucose) on subjective sensations of appetite and food intake in healthy men	16	24.7	21.1	≥3-d washout, 10–12-h fast, participant questionnaire measuring sleep habits and stress factors	VAS (2 h)	Wheat bran cereal (Fiber One - General Mills, Spain)	90.0	770	TF: 41.0	━	↑							↓	N
								Wheatlets cereal (control)^c^	44.0	770	TF: 1.0										
Gentile et al (2015) (+)^64^	N	Examine the effects of RS alone and in combination with whey protein supplementation on energy expenditure, substrate utilization, and hunger and satiety sensations	24 (-8)	45.8	26.5	≥4-d washout, 12-h fast	VAS (3.15 h)	Resistant starch pancake	NR	1661	HDP from WMS: 40.0	━	━	━		NR		NR			N
								Waxy Maize Starch pancake (control)	NR	1661	WMS: 0.0										
Gonzalez-Anton et al (2015) (+)^65^	Y	Assess the glycemic index, glycemic load, insulinemic index, appetite ratings, and postprandial plasma concentrations of gastrointestinal hormones after the consumption of 5 different most-common breads consumed in Spain	23 (-1)	25.0	23.3	≥1-wk washout, ≥10-h fast, menstrual cycle phase	VAS (3 h)	Whole-meal bread	134	1360	TF: 10.6	━	━	━				━		━	N
								White bread (control)^c^	95	1130	TF: 3.9										
Hamedani et al (2009) (+)^66^	N	Compare the effects of a high-insoluble- fiber cereal with a low-fiber cereal on food intake, subjective measures of appetite, and glucose levels in healthy individuals	32	20.0-26.0	20.5-24.5	1-wk washout, 10–12-h fast, menstrual cycle phase, participant questionnaire measuring sleep habits and stress factors	VAS (3 h)	Wheat bran cereal (Fiber One)	60.0	504	TF: 28.0 IF: 2.6 SF: 2.0	━	⇈			━		━		⇊	N
								Corn flakes cereal (control)^c^	60.0	911	TF: 1.5 IF: 0.3 SF: 1.2										
Hartvigsen et al (2014A) (+)^67^	N	Compare the effects of dietary fiber and whole grain on glucose, hormone, and appetite responses in subjects with MetS	15	62.8	31.1	1-wk washout, 12-h fast	VAS (4.5 h)	Arabinoxylan bread	136.0	1276	TF: 11.2 AX: 7.1 βG: 0.3 CE: 0.6 RS: 0.7	⇊	⇈	⇈				⇊		━	N
								β-Glucan bread	133.0	1108	TF: 13.4 AX: 2.6 βG: 4.2 CE: 4.2 RS: 1.4	⇊	↑	⇈				⇊		━	
								Rye kernel bread	147.0	1040	TF: 12.2 AX: 6.1 βG: 1.5 CE: 1.4 RS: 1.1	⇊	⇈	⇈				⇊		━	
								Wheat bread (control)^c^	107.0	1088	TF: 2.9 AX: 1.1 βG: 0.2 CE: 0.4 RS: 0.3										
Hartvigsen et al (2014B) (+)^68^	N	Determine the effect of isolated arabinoxylan alone or in combination with whole-grain rye kernels on postprandial glucose, insulin, free fatty acids, GI hormones, short-chain fatty acids, and appetite sensations in subjects with MetS	15	63.5	31.3	1-wk washout, 12-h fast	VAS (4 h)	Arabinoxylan semolina porridge	601.0	1210	TF: 10.2 AX: 3.5 AXOS: 0.8 RS: 1.1	↓	↑	↑				↓			N
								Arabinoxylan and rye kernel semolina porridge	435.0	1093	TF: 11.5 AX: 4.4 AXOS: 0.2 RS: 1.2	⇊	↑	↑				↓			
								Rye kernel semolina porridge	452.0	1001	TF: 12.7 AX: 4.7 RS: 1.4	↓	↑	↑				↓			
								Semolina porridge (control)	525.0	1005	TF: 5.7 AX: 0.9 RS: 1.8										
Heinonen et al (2007) (+)^69^	N	Ascertain whether circulating ghrelin is affected differently by 2 varieties of whole-grain breads known to catalyze low or high insulin responses in obese subjects with MetS	8	55.6	33.7	1-wk washout, 12-h fast	VAS (2 h)	Whole-grain rye bread	113.0	1093	TF: 10.2	⇊		⇈							N
								Wheat bread (control)^c^	125.0	1260	TF: 7.0										
Holt et al (2001) (+)^70^	N	Determine the effects of commercially available breads differing in their nutrient composition, energy density, and textural qualities on satiety index scores and subsequent ad libitum EI	10	23.5	22.1	1-wk washout, ≥10-h fast	VAS (2 h)	Coarse white bread	135.4	1019	TF: 15.9		↑	⇈						⇊	N
								High-fiber wheat bread	148.2	992	TF: 33.5		↑	⇈						⇊	
								Wonder White bread (control)	90.1	979	TF: 1.8										
Hughes et al (2022) (+)^71^	Y	Investigate the effects of RS type 2 from wheat on perceived appetite sensations and associated gastrointestinal hormones	30 (-7)	53.9	26.5	2-wk washout, 12-h fast	VAS (3.5 h)	Resistant starch type 2 wheat roll^f^	NR	3264	TF: 19.7 RS: 9.6 IF: 11.8 SF: 7.8	━	━			━		━			N
								Wheat roll (control)	NR	3301	TF: 4.7 RS: 1.8 IF: 2.3 SF: 2.4										
Isaksson et al (2009) (+)^72^	Y	Compare the satiating effect of isoenergetic breads and investigate the dose-response effect of rye bran and intermediate rye fraction	Study 1: 16 Study 2: 16 (-3)	Study 1: 35.0 Study 2: 38.0	Study 1: 22.0 Study 2: 23.0	6–8-d washout, 12-h fast, participant food diary kept prior to test day, participant compliance form	VAS (8 h)	Rye bran bread	133.0	1090	TF: 14.5	⇊		⇈		⇊					N
								Intermediate rye fraction bread	120.0	1090	TF: 6.5	⇊		⇈		⇊					
								Sifted rye bread	100.0	1090	TF: 3.5	⇊		⇈		⇊					
								Wheat bread (control)	98.0	1090	TF: 1.5										
								Rye bran bread (8 g DF)	121.0	1090	TF: 9.0	━		⇈		━					N
								Rye bran bread (5 g DF)	114.0	1090	TF: 6.0	━		⇈		━					
								Intermediate rye fraction bread (8 g DF)	126.0	1090	TF: 8.5	━		━		━					
								Intermediate rye fraction bread (5 g DF)	123.0	1090	TF: 6.0	━		⇈		━					
								Wheat bread (control)	98.0	1090	TF: 1.5										
Isaksson et al (2011) (+)^25^	Y	Investigate if variation in rye grain structure affects perceived appetite sensations	24 (-3)	25.0	22.7	5–15-d washout, 12-h fast, measurement of movement (step count), participant compliance form	VAS (8 h)	Milled rye kernel bread	158.0	1650	TF: 13.0 AX: 4.4 AG: 0.2 βG: 1.0 CE+RS : 3.7 FRU: 1.6	↓		⇈		↓					N
								Whole rye kernel bread	156.0	1600	TF: 11.6 AX: 4.4 AG: 0.2 βG: 1.0 CE+RS : 2.6 FRU: 1.7	↓		⇈		⇊					
								Wheat bread (control)	144.0	950	TF: 5.5 AX: 1.6 AG: 0.2 βG: 0.3 CE+RS : 1.4 FRU: 0.5										
Johansson et al (2015) (+)^73^	N	Investigate if whole-grain rye crisp bread compared with refined-wheat crisp bread produces beneficial effects on appetite and postprandial insulin response	23 (-2)	60.1	23.8	≥6-d washout, 12-h fast	VAS (4.5 h)	Yeast-fermented whole-grain rye crisp bread	60.0	867	TF: 18.3 AX: 8.6 AG: 0.2 βG: 2.1 CE+RS : 2.5 FRU: 2.6	⇊	⇈			━					N
								Yeast-fermented refined wheat crisp bread (control)^c^	52.0	867	TF: 6.0 AX: 2.5 AG: 0.2 βG: 0.1 CE+RS : 1.4 FRU: 0.4										
Karalus et al (2012) (+)^74^	N	Test the satiating properties of 4 isolated fibers added to chocolate crisp bars	22	25.0	23.7	≥1-wk washout, 12-h fast, menstrual cycle phase, adherence to low-fiber diet prior to test, carryover effects balanced using a Williams Latin Square Design	VAS (3 h)	Resistant wheat starch chocolate crisp bar	83.0	1604	TF: 11.7	━	━	━						━	Y^g^
								Soluble corn fiber chocolate crisp bar	83.0	1609	TF: 11.1	━	━	━						━	
								No-added-fiber chocolate crisp bar (control)	83.0	1709	TF: 2.4										
Klosterbuer et al (2012) (+)^75^	Y	Determine the effects of 3 novel fibers on satiety responses and serum parameters	20	29.8	23.0	≥3-wk washout, 12-h fast, menstrual cycle phase, adherence to low-fiber diet	VAS (2 h)	Soluble corn fiber meal^h^	NR	2592	TF: 27.8	━	━	━				━		━	N
								Resistant starch meal	NR	2475	TF: 27.2	━	━	━				━		━	
								Low-fiber meal (control)	NR	2484	TF: 2.8										
Korczak et al (2014) (+)^76^	Y	Determine differences in satiety response to 3 tests containing either 10 g oat bran, 10 g barley bran, and a low fiber condition consumed at dinner and breakfast	42	25.5	21.5	1-wk washout period, ≥12-h fast, menstrual cycle phase	VAS (4 h)	Barley bar	98.0	1932	TF: 10.0	━	━	━				━		━	N
								Oat bar	103.0	1940	TF: 10.0	━	━	━				━		━	
								Low-fiber bar (control)	103.0	1915	TF: 3.0										
Kristensen et al (2010) (+)^46^	N	Assess the effect of isoenergetic meals containing whole-meal wheat breads and pasta in comparison to similar refined wheat varieties on postprandial glycemia, appetite sensations, and ad libitum EI	16	24.1	21.7	Maximum 2 tests/wk, conversation controlled among participants	VAS (3 h)	Whole-grain wheat bread	146.3	2000	TF: 11.7	⇊	⇈	⇈				⇊		━	N
								Refined wheat bread (control)^c^	119.3	2000	TF: 3.6										
								Whole-grain wheat pasta	83.6	2000	TF: 5.0	X	X	━				X		━	N
								Refined wheat pasta (control)^c^	72.3	2000	TF: 2.2										
Mohr et al (2021) (+)^77^	Y	Establish the effects of pancake meals containing non-RS constituents or RS type 4 meals with and without greater protein contents on postprandial thermogenesis, fuel utilization, satiety scores, and gastro-entero-pancreatic hormones	8	49.0	28.2	≥4-d washout, 12-h fast	VAS (3.5 h)	Resistant starch pancake	NR	1661	RS from WMS: 40.0	⇊	X	⇊		━		NR			N
								Waxy maize starch pancake (control)	NR	1661	TF: 0.0										
Peters et al (2009) (+)^78^	Y	To determine the effect of isoenergetic meal-replacement bars containing fructo-oligosaccharide, β-glucan, or both on appetite sensations and food intake over 2 consecutive days	21 (-3)	52.8	25.9	1-wk washout, 10-h fast	VAS (4 h)	Barley breakfast bar	57.0	819	TF: 4.1 βG: 1.2 FOS: 1.2	━	━	━		━		━		━	N
								Breakfast bar (control)	56.0	811	TF: 2.5 βG: 0.3 FOS: 1.2										
Pletsch et al (2022) (+)^79^	Y	Assess the effect of whole-grain wheat compared with refined wheat milled products on postprandial glycemia, gastric emptying, and subjective appetite sensations	16	26.6	22.2	1-wk washout, 10-h fast	VAS (4 h)	Whole-grain wheat flour porridge	93.8	1113	TF: 9.6	━	━								N
								Reconstituted whole-grain wheat flour w/coarse bran porridge	72.8	1155	TF: 9.6	━	━								
								Reconstituted whole-grain wheat flour w/fine-bran porridge	72.8	1155	TF: 9.6	━	━								
								Refined grain flour porridge (control)^c^	78.9	1033	TF: 0										
Rebello et al (2013) (+)^80^	N	Compare the satiety effect of oatmeal with a popular ready-to-eat breakfast cereal when either was consumed as a breakfast meal	48 (-2)	34.1	26.1	1-wk washout, 10-h fast, menstrual cycle phase, meal time supervision	VAS (4 h)	Quaker Instant Oatmeal	66.8	1050	TF: 6.7 SF: 3.3 βG: 2.6	⇊	⇈	⇈		⇊		⇊			N
								Honey Nut Cheerios cereal (control)^c^	63.6	1050	TF: 4.5 SF: 1.8 βG: 1.7										
Rebello et al (2014) (+)^81^	N	Examine the effect of 2 types of oatmeal and an oat-based ready-to-eat breakfast cereal (RTEC) on appetite sensations, and assess differences in meal viscosity and β-glucan characteristics among the cereals	48 (-10)	29.8	27.1	3-d washout, 10-h fast, menstrual cycle phase, meal time supervision	VAS (4 h)	Quaker Instant Oatmeal	40.0	630	TF: 4.0 SF: 2.0 βG: 1.6	⇊	⇈			⇊		⇊			N
								Quaker Old Fashioned Oatmeal	40.0	630	TF: 4.0 SF: 2.0 βG: 1.6	↓	↑			↓		⇊			
								Honey Nut Cheerios cereal (control)^c^	40.0	630	TF: 2.7 SF: 1.1 βG: 0.0										
Rebello et al (2016) (+)^82^	Y	Compare the effect of 2 oat-based breakfast cereals on subjective appetite sensations and food intake	48 (-1)	32.5	24.9	1-wk washout, ≥10-h fast, meal time supervision	VAS (4 h)	Quaker Instant Oatmeal	66.8	1046	TF: 6.7 SF: 3.3 βG: 2.7	⇊	⇈			⇊		⇊		⇊	N
								Honey Nut Cheerios cereal (control)^c^	63.6	1046	TF: 4.5 SF: 1.8 βG: 1.7										
Rosén et al (2009) (ø)^83^	N	Explore the mechanism for a reduced postprandial insulin demand with rye products	12 (-1)	25.3	23.1	1-wk washout, 10-h fast	VAS (3 h)	Rye bran bread	141.7	NR	TF: 12.3 SF: 2.0 IF: 10.3			⇈							N
								Whole-grain rye bread	123.4	NR	TF: 9.6 SF: 2.8 IF: 6.8			↑							
								Endosperm rye bread	106.2	NR	TF: 6.7 SF: 2.5 IF: 4.2			↑							
								White wheat bread (control)	101.1	NR	TF: 1.8 SF: 0.8 IF: 1.0										
Rosén et al (2011A) (+)^84^	N	Evaluate the mechanism through which rye products may reduce postprandial insulin demand and to explore potential appetite-regulating properties	10	26.0	22.6	1-wk washout, 10-h fast	VAS (4.5 h)	Whole-grain rye bread	163.4	1078	TF: 26.3 IF: 19.8 SF: 4.0 RS: 2.5	⇊	⇈			↓				━	N
								Endosperm rye bread	134.8	1033	TF: 17.3 IF: 11.9 SF: 4.0 RS: 1.4	⇊	⇈			⇊				━	
								White wheat bread (control)	124.0	1015	TF: 6.0 IF: 4.4 SF: 0.5 RS: 1.1										
Rosén et al (2011B) (+)^85^	N	Investigate the effect of 5 rye grain varieties as well as a commercial blend of rye grown in Sweden on postprandial insulin, glucose, and subjective satiety	20 (-1)	26.7	22.2	1-wk washout, 10-h fast	VAS (3 h)	Amilo whole-grain rye bread	154.9	NR	TF: 12.6 IF: 9.0 SF: 3.6	↓	━			↓					N
								Evolo whole-grain rye bread	152.6	NR	TF: 13.3 IF: 9.3 SF: 4.0	⇊	⇈			↓					
								Kaskelott whole-grain rye bread	154.1	NR	TF: 13.8 IF: 10.0 SF: 3.8	↓	↑			↓					
								Picasso whole-grain rye bread	153.9	NR	TF: 13.4 IF: 9.7 SF: 3.7	↓	↑			↓					
								Vicello whole-grain rye bread	148.9	NR	TF: 13.1 IF: 10.1 SF: 3.0	↓	⇈			↓					
								Commercial whole-grain rye bread	157.6	NR	TF: 13.9 IF: 10.6 SF: 3.3	⇊	↑			⇊					
								White wheat bread (control)	125.9	NR	TF: 2.8 IF: 2.4 SF: 0.4										
Schroeder et al (2009) (+)^86^	Y	Compare the effect of whole-grain high-fiber barley, whole-grain wheat, and refined rice-based foods on satiety and EI	47 (-3)	31.0	23.0	1-wk washout, 10-h fast	VAS/SLIM^i^ (4 h)	Sustagrain barley snack mix	30.0	502.1	TF: 6.0 IF: 3.6 SF: 2.4	⇊	⇈			━		━		━	N
								Whole-wheat snack mix	30.0	460.2	TF: 2.0 IF: 1.8 SF: 0.2	━	⇈			━		━		━	
								Refined-rice snack mix (control)^b^	30.0	502.1	TF: 0.0										
								Sustagrain barley cereal	56.0	920.5	TF: 12.0 IF: 7.2 SF: 4.8	⇊	⇈			━		━		━	N
								Whole-wheat cereal	56.0	836.8	TF: 5.0 IF: 4.4 SF: 0.6	━	⇈			━		━		━	
								Refined-rice cereal (control)^c^	56.0	878.6	TF: 1.0 IF: 0.9 SF: 0.1										
Stefoska-Needham et al (2016) (+)^87^	Y	Test the effects of 3 different ready-to-eat whole-grain sorghum flaked breakfast biscuits on appetite responses	40	29.4	23.4	≥3-d washout, 12-h fast, menstrual cycle phase	VAS (4 h)	Brown sorghum flaked breakfast cereal	50.0	765	TF: 4.9	⇊	⇈	⇈		⇊				━	N
								Red sorghum flaked breakfast cereal	50.0	775	TF: 3.9	⇊	⇈	⇈		⇊				━	
								White sorghum flaked breakfast cereal	50.0	776	TF: 3.7	⇊	⇈	⇈		⇊				━	
								Wheat flaked breakfast cereal (control)	50.0	754	TF: 4.8										
Stewart et al (2018) (+)^47^	Y	Assess the postprandial glucose response, postprandial satiety, and gastrointestinal tolerance after consumption of a high-fiber scone containing a novel RS4 or a low-fiber control scone without RS4	35 (-1)	46.2	26.1	1-wk washout, menstrual cycle phase	VAS (3 h)	Resistant starch fiber scone	84.1	1130	TF: 14.9	⇊	━			⇊		━			N
								Control scone (control)	83.9	1372	TF: 4.0										
Vitaglione et al (2009) (+)^88^	N	Evaluate the effect of barley β-glucans on acute appetite and on satiety-related hormones	14	23.9	22.9	1-wk washout, ≥10.5-h fast, menstrual cycle phase, physical or psychological discomfort	VAS (3 h)	β-Glucan–enriched bread	100.0	1092	TF: 4.4 βG: 3.0	⇊	⇈	⇈						⇊	N
								Wheat flour bread (control)	93.0	1084	TF: 1.4 βG: 0.0										
Vuholm et al (2014) (+)^89^	Y	Investigate whether appetite sensations and EI is affected by the addition of dietary fibers to sausages	25 (-3)	25.2	23.2	≥3-d washout, 12-h fast	VAS (4 h)	Wheat bran sausage meal	382.0	2902	TF: 10.4	⇊	⇈	⇈				⇊		━	N
								Rye bran sausage meal	388.0	2875	TF: 9.8	⇊	⇈	⇈				⇊		━	
								Wheat flour sausage meal (control)	373.0	2753	TF: 4.4										
Weickert et al (2006) (ø)^90^	Y	Investigate effects of purified insoluble cereal fibers on postprandial PYY and ghrelin responses as well as satiety ratings	14	23.6	21.3	1-wk washout, 10-h fast	VAS (5 h)	Oat fiber bread	133.0	1800	TF: 10.6	━									N
								Wheat fiber bread	131.0	1012	TF: 10.5	↓									
								White bread (control)	103.0	1800	TF: 2.9										
Willis et al (2009) (+)^91^	Y	Compare a low-fiber muffin with a variety of high-fiber muffins containing different fiber types on their effects on satiety	20	26	22.9	1-wk washout, 12-h fast, menstrual cycle phase	VAS (3 h)	Corn bran muffin	99.0	729	TF: 9.6 IF: 9.5 SF: 0.5	⇊	⇈	⇈				⇊			N
								Barley β-glucan fiber muffin	96.0	733	TF: 9.4 IF: 5.3 SF: 4.0	↓	↑	↑				↓			
								Resistant starch muffin	92.0	729	TF: 8.0 IF: 7.9 SF: 0.1	⇊	⇈	↑				⇊			
								Low-fiber muffin (control)	76.0	745	TF: 1.6 IF: 1.2 SF: 0.4										
Wolever et al (2020) (+)^48^	Y	Determine the effect of altering the amount or molecular weight and viscosity of oat β-glucan in a breakfast meal on the primary endpoint of food intake at a subsequent meal	28 (-5)	33.1	24.8	≥5-d washout	VAS (3 h)	Oatmeal + 10.1 g oat bran	336.0	1582	TF: 9.3 βG: 4.0	⇊	━			⇊		━		━	N
								Oatmeal + 3.0 g oat bran	332.0	1573	TF: 5.6 βG: 2.0	━	↓			━		━		━	
								Cream of rice (control)	324.0	1582	TF: 1.4 βG: 0.0										
Ye et al (2015) (+)^92^	Y	Assess the effect of Fibersol-2 (Fibersol, USA), a water-soluble, non-viscous and highly digestion-resistant maltodextrin on hunger and satiety as well as gut satiety factors in healthy humans	19 (-1)	36.0	25.0	1-wk washout, 13-h fast	VAS (4 h)	Tea + 10 g Fibersol-2	NR	NR	TF: 10.0	⇊	↑	⇈				↓			N
								Tea + 5 g Fibersol-2	NR	NR	TF: 5.0	━	━	━				━			
								Control tea (control)	NR	NR	TF: 0.0										
Zamaratskaia et al (2017) (+)^93^	Y	Determine the effect of consuming sourdough-fermented and unfermented rye crispbread on self-rated appetite, postprandial glucose, and insulin response in healthy subjects	24	30.0	23.0	≥6-d washout, 10-h fast	VAS (6 h, 40 min)	Unfermented rye crispbread	59.8	830	TF: 11.7	↓	↑		⇊						N
								Sourdough-fermented rye crispbread	59.4	821	TF: 9.5	⇊	↑		⇊						
								Yeast-fermented refined wheat crispbread (control)	52.0	880	TF: 2.9										

Interpretation of quality rating: +, positive, ø, neutral. Appetite sensation key: ↑, increase; ⇈, statistically significant increase; ━, no significant difference in effect; ↓, decrease; ⇊, statistically significant decrease; X, unable to be determined; 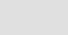, control; ▪▪▪, outcome not measured.

a Sample size refers to the total number of participants who successfully completed the study.

b Total fiber reported where specific fiber source(s) not specified.

c Deemed as the control by the authors of this systematic review due to lower fiber content.

d Participant withdrawal due to worsening glycemic control.

e Test products were served with a butter and cheese portion.

f Test products were served with egg, cheese, and turkey sausage.

g All bars produced more bloating and flatulence compared with the control.

h Test meals consisted of a muffin and hot cereal made using specified fiber type.

i A modified version of the VAS scale was used (SLIM).

Abbreviations: AG, arabino-galactan; AX, arabinoxylan; AXOS, arabinoxylan oligosaccharides; BAR, β-glucan enriched barley; BMI, body mass index; CE, cellulose; DF, dietary fiber; DTE, desire to eat; EI, energy intake; FOS, fructo-oligosaccharides; FRU, fructan; GI, gastrointestinal; GIT, gastrointestinal tract; HAW, high-amylose wheat; HDP, hydroxypropyl di-starch phosphate; IF, insoluble fiber; LAW, low-amylose wheat; MetS, metabolic syndrome; N, no; NR, not reported; PFC, prospective food consumption; PMP, pearl millet porridge; PYY, peptide YY; RS, resistant starch; SF, soluble fiber; SLIM, satiety-labelled intensity magnitude; SOP, Scottish oats porridge; T2DM, type 2 diabetes mellitus; TF, total fiber; VAS, visual analog scale; WMS, waxy maize starch; Y, yes; βG, β-glucan.

### Characteristics of Publications

The studies were conducted in 13 different countries, resulting in a heterogenous collection of study populations from the United States (15 studies),^47^^,^^60^^,^^64^^,^^71^^,^^74–77^^,^^79–82^^,^^86^^,^^91^^,^^92^ Sweden (8),^25^^,^^62^^,^^72^^,^^73^^,^^83–85^^,^^93^ Denmark (5),^46^^,^^61^^,^^67^^,^^68^^,^^89^ Australia (4),^54^^,^^55^^,^^70^^,^^87^ Italy (4),^53^^,^^56^^,^^59^^,^^88^ United Kingdom (3),^50^^,^^51^^,^^57^ and Canada (3),^48^^,^^63^^,^^66^ and 1 study each in Finland,^69^ Germany,^90^ Ireland,^58^ Japan,^52^ Netherlands,^78^ and Spain.^65^ The key characteristics of participants included an age range of 18–73 years and mean BMI of 24.3 kg/m^2^ (16.6–38.7 kg/m^2^). Various cereal fiber types included rye (*n* = 32), wheat (*n* = 19), oats (*n* = 13), barley (*n* = 8), sorghum (*n* = 3), millet (*n* = 3), corn (*n* = 1), and added exogenous fiber resistant starch (RS) (*n* = 12), arabinoxylan from wheat (*n* = 2), and soluble corn fiber (SCF) (*n* = 2). Intervention arms were typically compared with a refined version of the same product—for example, a refined wheat, corn, rice, or waxy maize starch control. The full results are summarized in Table S2. All comparisons had a suitable test food and control; however, there was variation in the test foods, portion size, and fiber amount. The minimum reported fiber dose to produce a favorable statistically significant effect was 2.0 g of total fiber^53^ and the maximum dose was 40.0 g of total fiber.^77^ Test foods of solid or semisolid form comprised 95 of the 98 included study interventions, with the remaining in a beverage format.

### Study Quality

Nearly all studies (94%) were awarded a positive rating, with the remaining 6% receiving a neutral rating; therefore, no studies received a negative quality rating. Aspects impacting quality outcomes included a lack of detail pertaining to the study population, degree of comparability between study groups, and little description on the method of handling withdrawals.

### Effect of Cereal Fiber on Acute Subjective Measures of Satiety

The majority of comparisons between higher vs lower cereal fiber intervention arms (*n* = 98) displayed a high rating of consistency for satiety as indicated by a score of 75% or greater (Table 4). Hunger, fullness, and desire to eat scored a moderate consistency rating (60%–74%), with prospective food consumption and ad libitum energy intake scoring a low consistency rating (<60%). Although 4 of the 5 appetite sensations were rated as low or moderate consistency, this was not necessarily indicative of a negative association. Rather, positive effects were observed in the majority of studies, although outcomes did not reach statistical significance in some studies.

**Table 4.** Consistency Ratings for Appetite Sensations and Ad Libitum Energy Intake

Outcomes	No. of intervention arms that assessed effect	**Consistency rating on direction of favorable effect in higher quality studies** ^a^
Hunger	89	67.4%
Fullness	76	69.4%
Satiety	67	75.4%
Desire to eat	52	68.0%
Prospective food consumption	52	57.1%
Overall appetite effect^b^	—	67.6%
Ad libitum energy intake	49	25.6%

a Interpretation of consistency rating: ≥75.0% denotes a high rating of consistency on direction of effect, 60.0%–74.0% denotes a moderate consistency rating, and <60.0% denotes a low consistency rating.^41^

b The average of the 5 appetite sensations (hunger, fullness, satiety, desire to eat, and prospective food consumption) was used to determine overall appetite effect.

The majority of intervention arms comprised higher-cereal-fiber products derived from rye, wheat, and oats (67.4%), with the remaining portion comprising barley, sorghum, millet, and corn as well as the extracted fiber arabinoxylan and functional fibers RS and SCF (32.6%). The minimum to maximum fiber dose delivered through a single intervention for each cereal fiber included 5.0–26.3 g for rye, 2.0–41.0 g for wheat, 4.0–13.4 g for oats, 3.0–12.0 g for barley, 3.7–4.9 g for sorghum, 6.8–13.8 g for millet, 10.2–11.2 g for arabinoxylan, 9.6 g for corn, 5.0–40.0 g for RS, and 11.1–27.8 g for SCF. Approximately 9.5% of total intervention arms comprised a combination of cereal fibers—for example, added arabinoxylan from wheat, added to other grains; however, effects of these cereals on appetite outcomes were not independently assessed. Causality between the low-frequency cereal fibers (sorghum, millet, arabinoxylan, corn, and SCF) and satiety could not be determined due to few intervention arms. It is noteworthy that these cereal fibers still indicated a high rating of consistency (with the exception of SCF), suggesting an overall positive effect on appetite outcomes compared with a low-fiber control (Table 5).

**Table 5.** Consistency Ratings on Direction of Favorable Effect Among High-Quality Studies for Varying Cereal Fiber Sources and Their Associated Effect on Each Appetite Sensation and Ad Libitum Energy Intake

	Source of cereal fiber																																									
	Rye								**Wheat** ^a^						Oats						Barley						Sorghum		Millet		Arabinoxylan		Corn		Resistant starch						Soluble corn fiber	
	Total (*n* = 32)		Whole grain (*n* = 18)		Added fiber (*n* = 12)		Unknown (*n* = 2)		Total (*n* = 19)		Whole grain (*n* = 12)		Added fiber (*n* = 7)		Total (*n* = 13)		Whole grain (*n* = 3)		Added fiber (*n* = 10)		Total (*n* = 8)		Whole grain (*n* = 4)		Added fiber (*n* = 4)		Whole grain (*n* = 3)		Whole grain (*n* = 3)		Added fiber (*n* = 2)		Added fiber (*n* = 1)		**Total (*n* = 12)**		Whole grain (*n* = 1)		Added fiber (*n* = 11)		Added fiber (*n* = 2)	
	*n*	%	*n*	%	*n*	%	*n*	%	*n*	%	*n*	%	*n*	%	*n*	%	*n*	%	*n*	%	*n*	%	*n*	%	*n*	%	*n*	%	*n*	%	*n*	%	*n*	%	*n*	%	*n*	%	*n*	%	*n*	%
Appetite sensations																																										
Hunger	29	82.8	17	94.1	10	60.0	2	100.0	14	23.1	10	20.0	6	20.0	13	83.3	3	100.0	10	77.7	8	75.0	4	75.0	4	75.0	3	100.0	3	100.0	2	100.0	1	100.0	10	40.0			10	40.0	2	0.0
Fullness	15	93.3	13	92.3	2	100.0			14	57.1	9	33.3	7	71.4	12	75.0	3	100.0	9	66.6	8	75.0	4	75.0	4	75.0	3	100.0	3	100.0	2	100.0	1	100.0	9	22.2			9	22.2	2	0.0
Satiety	16	93.8	8	100.0	11	88.8			10	55.6	7	33.3	3	100.0	8	87.5	1	100.0	7	85.7	6	66.6	2	50.0	4	75.0	3	100.0	3	100.0	2	100.0	1	100.0	10	50.0	1	100.0	9	44.4	2	0.0
Desire to eat	22	77.3	14	92.8	9	50.0			3	50.0	4	33.3			6	83.3	3	100.0	3	66.6	3	0.0	3	0.0			3	0.0	3	100.0					6	66.6	1	100.0	6	50.0		
Prospective food intake	6	83.3	3	66.6	1	100.0	2	100.0	9	22.2	6	16.6	2	50.0	12	75.0	3	100.0	10	60.0	6	33.3	4	25.0	2	50.0	0	NR	3	100.0	2	100.0	1	100.0	7	28.6			7	28.6	1	0.0
Overall appetite effect^b^		86.1		89.2		79.8		100.0		41.6		27.3		60.4		80.8		100.0		71.3		50.0		45.0		68.8		75.0		100.0		100.0		100.0		41.5		100.0		37.0		0.0
Secondary outcome																																										
Ad libitum energy intake	8	12.5	4	25.0	2	100.0	2	0.0	11	45.5	6	16.6	5	80.0	6	16.6			6	16.6	8	37.5	4	25.0	4	50.0	3	0.0	1	0.0	1	0.0			3	33.3			3	33.3	2	0.0

Interpretation of consistency rating: ≥75.0% denotes a high rating of consistency on direction of effect, 60.0%–74.0% denotes a moderate consistency rating, and <60.0% denotes a low consistency rating.^41^

a Wheat sources derived from whole wheat (*n* = 12), wheat bran (*n* = 5), and wheat fiber (*n* = 2).

b The average of the 5 appetite sensations (hunger, fullness, satiety, desire to eatm and prospective food consumption) was used to determine overall appetite effect.

Abbreviation: NR, not reported.

Intake of rye products with a higher-fiber content showed the highest rating of consistency on overall appetite effect (86.1%), followed by intake of higher-fiber oat products (80.8%) when tested against lower-fiber counterparts. Intake of whole-grain products from rye- and oat-based sources produced a greater rating of consistency compared with their added-fiber counterparts. In contrast, intake of higher-fiber wheat varieties and barley products demonstrated a low rating of consistency on overall appetite effect when tested against a low-fiber control (41.6% and 50.0%, respectively). Similarly, low ratings of consistency were observed with RS and SCF (41.5% and 0.0%, respectively). The secondary analysis of 46 comparisons measuring the effect of higher cereal fiber intake on ad libitum energy intake revealed a low consistency rating, suggesting a weak effect on decreasing ad libitum energy intake. It is noteworthy that, when examining differences between consumption of fiber naturally occurring in foods and added/extrinsic fiber types, a high consistency rating for ad libitum energy intake was found among higher intakes of added-fiber types for rye and wheat interventions (100.0% and 80.0%, respectively).

## DISCUSSION

### Principal Findings

The results of this review support the findings of existing literature, which substantiate that the consumption of higher-cereal-fiber–containing foods in comparison to lower-cereal-fiber–containing foods results in positive appetite outcomes—in particular, increased satiety, which may have the propensity to contribute to a reduction in cumulative energy intake and subsequent weight management. Subgroup analysis of cereal fiber sources shows apparent differences in appetite outcomes, potentially alluding to differences in physicochemical properties between various cereal fibers and their resulting outcome on appetite sensations. Furthermore, this analysis uncovered an overall consistently poor effect on ad libitum energy intake across all cereal fibers, with the exception of added fibers from rye- and wheat-based sources. It appears that the cereals containing a greater proportion of soluble fiber (such as rye, oats, and barley) rather than insoluble fiber (such as whole-grain wheat) provide far greater effects on increasing acute satiety; however, this could not be confirmed as the majority of studies did not include a defined measurement of solubility.

The presence of discrepancies in individual appetite outcomes and ad libitum energy intake between varying cereal fiber sources warrants consideration of other competing factors that may also possess appetite-mediating effects. These include the cereal variety and portion size, food matrix, solubility, composition of test food and cereal fiber types, and other relevant properties including energy density, macronutrient content, carbohydrate uptake, and the presence of polyphenols. In addition, although we ensured that a reasonable wash-out period was part of included studies, only a limited number of studies recorded diet immediately before a test day or habitual diet (Table 3). The recording of background dietary information is critical in all dietary intervention studies, where ideally, the only dietary difference is the food being tested.^94^ While this review contributes to the existing evidence base that substantiates a positive correlation between higher cereal fiber consumption and satiety, establishing a recommended dose connecting cereal fiber with increased satiety may be difficult given the heterogeneity in interventions, fiber ratios, and methods of processing observed in the existing literature, which may all have the propensity to modulate appetite.

### Cereal Fiber Types and Effects on Appetite and Ad Libitum Energy Intake

#### Rye

Higher-cereal-fiber products from rye resulted in favorable effects on appetite outcomes, in particular an increase in fullness and satiety. This is synonymous with the findings of various studies assessing the satiating properties of rye among healthy adult populations. Such studies have uncovered a link between level of processing and degree of satiety, with minimally refined products, such as rye kernels and porridges, possessing a stronger effect on satiety than milled rye flours.^95^ One randomized crossover study tested the effects of several differentially processed rye or wheat tests consumed at breakfast, including refined-grain wheat, refined-grain endosperm rye bread, whole-grain rye bread, whole-grain wheat kernels, and whole-grain rye kernels.^84^ It was found that the rye tests favorably increased satiety in the acute postprandial phase, with rye kernels having the most significant effect on satiety in an acute sense as well as in the instance of a second meal as evidenced by lower ad libitum intake and the reported VAS scores compared with the consumption of the refined-wheat bread control.^84^ Similarly in this review, intake of whole-grain rye products scored a higher rating of consistency compared with intake of products containing highly refined or added rye fibers. These results may be explained by inherent differences in the rate of uptake between minimally and heavily refined grains as a result of differences in digestive and absorptive capacity.^96^

The consumption of higher-fiber rye products had a weak effect on ad libitum energy intake, which aligns with findings of previous studies indicating differences in subjective appetite measures and the absence of effects on energy intake.^46^^,^^97^^,^^98^ It has been proposed that meal volume and timing may contribute to such effects, as seen with 1 study that found a 16% reduction in energy intake after consuming rye kernels compared with refined wheat bread; however, the volume of meals differed by approximately 50%, which may explain its effect on satiety at the ad libitum meal.^84^ Although this review did not assess the direction of favorable effect according to the solubility properties of the included cereals, it is important to acknowledge the potential effect that this may have had on overall appetite outcomes and ad libitum energy intake. Noting that the ratio of soluble and insoluble fiber varies depending on cereal type, findings of this review remain consistent with existing evidence, which supports beneficial effects on acute appetite outcomes from the intake of foods containing a greater proportion of soluble fiber. Rye contains high amounts of soluble fiber in the form of arabinoxylan and β-glucan.^99^ These fibers have viscous properties and a propensity to accumulate larger amounts of water within the gastrointestinal tract (GIT), thereby increasing viscosity and abdominal distention and disrupting nutrient uptake kinetics, which stimulates satiety through hormonal, mechanical, and electrical signals.^62^^,^^81^

#### Wheat

Findings from this study suggest a weak effect of higher-fiber wheat products on overall appetite effect and other measures of satiety. Differences between wheat sources were found, with added-fiber types displaying a moderate rating of consistency on overall appetite effect compared with a low rating of consistency observed with whole-grain varieties. These findings differ from those of a recent systematic review investigating the effects of whole-grain intake compared with refined grain on appetite outcomes and energy intake. Sanders et al^100^ found that intake of higher-fiber whole-grain wheat products significantly reduced hunger and desire to eat while increasing fullness compared with refined-grain wheat products. However, subgroup analyses revealed differences in appetite sensations according to energy content and volume of test foods, both of which have the propensity to impact satiety. Matching interventions for energy intake and volume or energy intake alone showed no significant difference in appetite sensations between whole-grain and refined-grain products. Sanders et al^100^ also found that intake of higher-fiber whole-grain wheat was unable to produce a significant effect on ad libitum energy intake at a subsequent meal. This is in line with the findings of the present review, which observed a weak effect on ad libitum energy intake related to intake of higher-fiber wheat products.

It is possible that the weak effect observed between intake of higher-fiber wheat and appetite sensations may be due to differences in the physicochemical factors of wheat, including hydration and viscosity properties.^80^ Wheat is one of the most common forms of insoluble dietary fiber, which is known to decrease GIT transit time and relieve constipation related to bulking properties. Unlike soluble fibers, interaction with water occurs by means of hydrophobicity, and the effect of insoluble fibers on satiety is ambiguous.^86^ The available literature suggests that effects of insoluble fiber on satiety are associated with chronic consumption, which causes physiological adaptations of the GIT and an ability to undergo partial fermentation within the large intestine, catalyzing a subsequent shift in the neurohormonal appetite response.^66^ As a result, the effects of insoluble fiber have been found to transpire at a minimum 3 hours and until 15 hours following treatment.^28^ This may also provide a rationale for the substantially lower consistency rating observed between wheat and a soluble dietary fiber such as rye. However, 1 study found a significant reduction in appetite responses following the consumption of a meal abundant in insoluble fiber from grains, although favorable effects on appetite parameters were observed with a considerable dose of fiber equal to 33 g.^98^ It is important to note that the present review focused on acute satiety responses and hence outcomes may have been skewed towards fiber types with inherent short-term effects rather than any potential longer-term influence of insoluble fibers on appetite.

Differences in consistency ratings between whole-grain wheat and added-wheat-fiber interventions may be attributed to the amount of fiber provided in each test, with an average dose of 8.2 g for whole-grain interventions and 19.8 g for added-wheat-fiber interventions as well as the ability for wheat bran fiber to retain water to a certain extent, resulting in larger volume and increased gastric distension.^63^

#### Oats

Intake of higher-fiber oat products displayed a high rating of consistency for overall appetite effect, suggesting a favorable effect on measures of satiety. These findings are consistent with existing human studies assessing the acute effects of oat β-glucan on appetite sensations in the form of whole foods and β-glucan extracts. For example, in studies assessing the influence of oat-based breakfast cereals on appetite outcomes, a 1046-kJ serving of rolled oats containing 2.6 g of β-glucan increased subjective satiety compared with an isoenergetic oat-based breakfast cereal containing 1.7 g of β-glucan.^80^^,^^81^ Additionally, the rolled oats indicated increased meal viscosity (following initial oral and gastric digestion) compared with the oat-based cereal.^81^^,^^82^ It has been proposed that meal viscosity at the initial phase facilitates the commencement of signaling through orosensory stimulation to enact an overall effect on satiety.^20^ β-Glucan doses between 2.2 g and 5.5 g have been shown to increase satiety; however, dose effects on satiety are incongruous^80^^,^^81^^,^^101^^,^^102^ and the autonomous effects of β-glucan could not be assessed alone in this review due to the presence of other cereal fibers in foods within the interventions.

While the studies assessing the effect of higher-fiber oat–based products in the present review all included tests in a semisolid form, it is still important to consider the findings of the existing literature examining the influence of the food matrix on satiety response specifically related to β-glucan ingestion. In 1 study, participants were served various breakfast meals comprising biscuits and diluted orange juice.^103^ Approximately 4 g of β-glucan was added to some of the biscuits and juice drink. The β-glucan content of a meal was found to increase the viscosity, irrespective of the food form. In addition to enhanced perceptions of satiety due to the added β-glucan, the combination of fortified biscuits with the fortified juice drink resulted in the strongest impact on satiety. Furthermore, fortifying the juice with oat bran proved more effective in enhancing satiety compared with its addition to the biscuits.^103^ However, a comparison between solid forms, oat β-glucan, rye kernels, and arabinoxylan in bread found increased perceptions of satiety compared with a refined-wheat-bread control, although no effect was found on energy intake, which aligns with the findings of this review whereby higher intake of oat-based products showed a weak effect on ad libitum energy intake.^67^ Therefore, the food matrix may serve to mediate the influence of viscosity on satiety, as confirmed by the absence of effect in studies with interventions of a semisolid form.^102^^,^^104^^,^^105^

#### Barley, Sorghum, Millet, and Corn

A low rating of consistency was found for intake of higher-fiber barley products on overall appetite effect and ad libitum energy intake. The findings of this review add to the body of evidence, which suggests mixed effects of barley on satiety response and energy intake.^106–111^ Some studies indicate that barley increases satiety compared with a food containing no dietary fiber, with the effect appearing consistent across various genotypes of barley.^108^^,^^109^^,^^112^ The favorable effects observed with barley are believed to be attributed to the relatively high concentration of β-glucan (3%–7%), which is known to delay postprandial intestinal glucose absorption and extend satiety duration,^113^ although the type and amount of β-glucan and test food have differed between the studies, which therefore may reveal why the literature is inconclusive. In addition, there is also some relevance of dose, with potentially a minimum dose required.^53^^,^^88^ Viscosity of β-glucan is critical for its effects, as a function of both MW and solubility,^78^ and is significantly affected by isolation, purification, and extraction processes.^114^^,^^115^ Hence, additional studies examining the rheological behavior of test foods containing β-glucan under simulated gastrointestinal conditions may be needed to circumvent this problem.^78^

This review found that the intake of higher-fiber sorghum, millet, and corn-based products resulted in a high rating of consistency for overall appetite effect; however, no effect was found on ad libitum energy intake. It is not possible to comment on the true effects of these cereal fibers on satiety and ad libitum energy intake due to the lack of studies. However, it would be reasonable to consider the effect of other nutrients besides fiber on satiety, such as the higher level of polyphenolic content in sorghum compared with wheat,^87^ the intrinsically high protein content of millet,^116^ and the low digestible starch component in corn.^91^

#### Arabinoxylan, Resistant Starch, and Soluble Corn Fiber

The functional fibers RS and SCF as well as the added-fiber arabinoxylan from wheat were found to have a low consistency rating for overall appetite effect and ad libitum energy intake in this review. These results contradict the findings of a recent systematic review examining the effects of acute RS consumption on appetite, which found significant decreases in appetite ratings following RS ingestion.^117^ The authors suggest that such effects on appetite are determined by the type and dose of RS, and found that the appetite-suppressing effects were stronger when the dose was 25 g or greater or when type 2 RS was administered.^117^ This may explain the difference in findings of the present review, whereby the majority of studies assessing RS (83%) used RS types other than type 2 and the average dose administered was 18.3 g. Research pertaining to the mechanism of action for acute satiety related to RS consumption is unclear. Available literature suggests that certain doses and types of RS may increase the expression of glucagon-like-peptide 1 and peptide YY, thereby improving satiety^91^; however, further studies are needed to confirm this.

The literature pertaining to the effect of extracted arabinoxylan from wheat on appetite in humans is scarce. It is possible that the favorable effects on appetite observed in the few studies assessing arabinoxylan in this review were confounded by the higher energy and protein content of the tests compared with the control and hence further research is needed to assess the satiety influence of concentrated arabinoxylan. Similarly, the effect of SCF on satiety has not been well researched. The findings in this review pertaining to SCF are consistent with existing studies, which have found no effect of this fiber on appetite outcomes or energy intake.^74^^,^^75^

### Strengths and Limitations

A strength of this review was the inclusion of randomized crossover trials, which assume the highest level of evidence and greatly reduce the ensuing effects of confounders as each participant acts as their own control. Additional strengths include the use of rigorous methodology and validated tools, with much of the methodology conducted in duplicate, enabling the collection of the most pertinent literature with high methodological quality and minimal confounders and bias.

While the use of a VAS enabled valid and reliable measurement of a participant’s motivation to eat in that it is sensitive to experimental manipulation and is reproducible in various experimental regimens,^32^ it also lacks objectivity. It is important to acknowledge that subjective ratings of appetite are not an inevitable product of intrinsic physiological activities but rather the participant’s perception of their motivations and sensations, which are greatly influenced by inherent physiological processes, among other factors.^32^ Furthermore, qualitative research has indicated that sensations of mental hunger and satiety can overlap and, hence, the general constructs of hunger and satiety may not be accurately assessed using a scale of simple, polar opposites.^118^ Hence, a VAS may serve as a useful adjunct to objective measures of food and energy intake, such as evaluating concentrations of appetite-regulating hormones such as glucagon-like-peptide 1, peptide YY, cholecystokinin, leptin, and ghrelin.^119^

Appetite outcomes may be unfairly represented, with the majority of included studies evaluating sources of cereal fibers from rye, wheat, and oats and less from other cereal fiber sources such as barley, sorghum, millet, and corn. Consideration of other influencing factors on appetite, such as the nutrient composition of test foods, physicochemical properties, and any processing effects, was not directly analyzed in this review. Also, this review only considered acute effects, which may have skewed outcomes for fibers with longer effects. Hence, exploring these factors may provide clearer insight into any appetite-mediating associations and to ascertain whether a dose–response relationship exists. The available literature has predominantly addressed the acute effects of fiber on satiety; while this may provide practical benefit, sustained dietary behaviors are vital in the prevention of obesity. Therefore, human studies assessing the effects of fibers contained both naturally in foods and extracted or extrinsic cereal fibers over a longer duration will be imperative in understanding gut adaptations to types and doses of cereal fibers in the context of satiety signaling. Furthermore, additional research is needed to investigate whether gender, age, or disease state modulate the influence of cereal fiber on appetite outcomes as well as further exploration of their physicochemical characteristics and related effects.

## CONCLUSION

The evidence suggests that satiety and other related appetite sensations are favorably impacted by the ingestion of higher-cereal-fiber–containing (especially sourced from rye and oats) foods in comparison to a lower-fiber control. Overall, higher-cereal-fiber interventions improved appetite measures of hunger, fullness, satiety, and desire to eat compared with a lower-fiber control; however, a favorable effect was not obvious with ad libitum energy intake. These findings support existing research that enhanced satiety from habitual intake of cereal-based products containing high amounts of fiber may be a relevant mechanism in health outcomes noted with ingestion of these foods.

## Supplementary Material

nuaf083_Supplementary_Data

## Data Availability

All data extraction is provided. Screening records are available on request from the authors.
