# Supplementary material for: Cereal Fibers and Satiety: A Systematic Review
Source: Nutr Rev. 2025 Jul 11;84(1):47–68. doi: 10.1093/nutrit/nuaf083 (PMC12696374; doi:10.1093/nutrit/nuaf083)
Supplement: nuaf083_Supplementary_Data [file nuaf083_supplementary_data.zip › Supplementary Materials - Cereal fibres and satiety SR.docx]

**Supplementary Materials**

**Supplementary Table 1 –** Full search strategy for each database

| **Database** | **Keyword(s)** | **Subject term(s)** | **Search strategy** | **Filters applied** |
| --- | --- | --- | --- | --- |
| **CINAHL Plus** | (“edible grain” OR poaceae OR wholegrain OR “whole grain”)  AND  (satiety OR appetite) | (MH “cereals”) OR (MH “dietary fiber”)  AND  (MH “appetite”) OR (MM “satiation”) OR (MH “satiety response”) | "edible grain" OR (MH "Cereals") OR poaceae OR wholegrain OR "whole grain" OR (MH "dietary fiber")  AND  (MH "appetite") OR satiety OR appetite  AND  (MM "satiation") OR (MH "satiety response") | **Expanders:** Apply equivalent subjects  **Narrow by Language:** English  **Narrow by Subject Major:** cereals  **Search modes:** Boolean/Phrase |
|  |  |  |  |  |
| **Medline** | (“edible grain” OR cereals OR poaceae OR wholegrain OR “whole grain” OR “dietary fiber”)  AND  (appetite OR satiety) | (MM “cereals”)  AND  (MM “satiation”) OR (MH “satiety response”) | AB ("edible grain" OR cereals OR poaceae OR wholegrain OR "whole grain" OR "dietary fiber" AND appetite OR satiety OR MM cereals)   AND   MM "satiation" OR MH "satiety response" | **Expanders:** Apply equivalent subjects  **Narrow by Language:** English  **Narrow by Subject Major:**  dietary fiber, hunger, appetite, energy intake, obesity, feeding behavior, eating, satiety response, satiation,  **Search modes:** Find all my search terms |
| **Web of Science** | (“edible grain” OR cereals OR poaceae OR wholegrain OR “whole grain” OR “dietary fiber”)  AND  (appetite OR satiety OR satiation OR “satiety response) |  | (“edible grain” OR poaceae OR wholegrain OR “whole grain” OR “dietary fiber”(Title) OR cereals (Title)  AND  (appetite OR satiety (Title) OR satiation OR “satiety response (Abstract)) | **Document types:** Article  **Language:** English  **Research areas**: Nutrition and Dietetics |
| **Scopus** | (“edible grain” OR cereals OR poaceae OR wholegrain OR “whole grain” OR “dietary fiber”)  AND  (appetite OR satiety OR satiation OR “satiety response) | - | TITLE-ABS-KEY ("edible grain" OR cereals OR poaceae OR wholegrain OR "whole grain" OR "dietary fiber"   AND   appetite OR satiety OR satiation OR "satiety response") | **Document types:** Article  **Language:** English  **Exclude keywords: “**Animals”,  “Nonhuman”, “Animal Experiment”, “Rat”, “Animal Food”, “Rats” |

**Supplementary Table 2 –** Overview of included publications (n=48).

| **Author** | **Article title** | **Study location** | **Study objectives** | **Study design** | **Blinding** | **Population baseline characteristics** | **Inclusion / Exclusion Criteria** | **Fasting period (hrs)** | **Washout period** | **Study / VAS duration (hrs)** | **Total no. of test days** | **Confounders adjusted for at baseline** | **Intervention arms** | **Primary Outcome** | **Secondary Outcome** | **Ad libitum food intake** | **No. of drop outs** | **Adverse effects** |
| --- | --- | --- | --- | --- | --- | --- | --- | --- | --- | --- | --- | --- | --- | --- | --- | --- | --- | --- |
| **Alyami et al., 2019A (+)*** | Glycaemic, GI, hormonal and appetitive responses to pearl millet or oats porridge breakfasts: a randomised, crossover trial in healthy humans | United Kingdom | To investigate the effect of a pearl millet porridge (PMP) compared with a well-known Scottish oats porridge (SOP) on glycaemic, GI, hormonal and appetite responses | Randomised two-way crossover | Y | **Participants:**  n = 26 (m = 9, f = 17)  **Age:**  x̄ 28.5 yrs (SD 9.6)  **BMI:**  x̄ 23.4 kg/m^2^ (SD 3.2)  **Condition:**  NIL | **Inclusion:**  - 18–65 years  - Healthy  BMI ≥18 and ≤24·9 kg/m2  - Can give written informed consent  **Exclusion:**  - Using medication which interferes with study measurements  - Participation in another nutritional trial 3 months prior  - Not habitually consuming  3 meals/day  - Not willing to consume study foods  - Working night shifts  - Partaking in strenuous exercise for >10h/week  - Consumption of 21 alcoholic beverages in a typical week  - Adhering to a medically or self-prescribed diet 2 weeks prior to and during the study  - MRI contraindication  - Pregnancy or inability to lie flat  - Exceeding the scanner bed weight limit (120 kg) | ≥ 10 | 1 week | 5.5 / 2.5 | 2 | - 1 week wash out period  - ≥ 10 hr fast  - MRI conducted at baseline to verify stomach contents  - Habitual intake between tests  - Both interventions were prepared using the same methods (i.e., plain water) to mitigate effects on starch gelatinisation and the presence of macronutrient confounders | **Food:**  Scottish Oats Porridge **(Control)** vs. Pearl Millet Porridge (PMP)  **Amount:**  C: 620mL, 920kJ, 7.2g P, 42.0g CHO, 4.4g F  PMP: 620mL, 920kJ, 6.6g P, 44.4g CHO, 3.3g F  **Fibre dose:**  C: 8.0g TF, 3.1g IF, 4.9g SF, 2.9g βG  PMP: 9.4g TF, 6.4g IF, 3.0g SF, 1.6g βG | n = 26 (incl. in analysis)  Feelings of hunger, DTE and PFC ↓ from fasting baseline following consumption of C and PMP porridges returning to baseline 2 hrs later. Feelings of fullness and satisfaction ↑ post intervention returning to baseline after 2 hrs.  No sig. differences by iAUC/ANOVA between test porridges for individual appetite ratings (p > 0.05). Composite satiety scores for both test porridges were not statistically sig. (p = 0.708)  Hunger iAUC2h p = 0.271  Satisfaction iAUC2h p = 0.685  Fullness iAUC2h p = 0.412  DTE iAUC2h p = 0.812  PFC iAUC2h p = 0.985 | No sig. difference in EI from ad libitum meal following PMP and C consumption. EI from ad libitum meal (p = 0.328)  No sig. difference in recorded food intake for the remainder of the day between the two intervention arms (p > 0·05).  - Glucose  - Insulin  - Plasma GLP-1  - Plasma GIP  - Appearance of - gastric content  - Gastric volumes  - Plasma PYY | Tomato and mozzarella pasta bake (Tesco). 129 kcal/100 g, providing 5·5 g protein, 17·0 g CHO, 3·6 g fat and 3·0 g fibre.  2.25 hours post interventions @ BF. | 0 | NIL |
| **Alyami et al., 2019B**  **(+)** | Glycaemic, gastro- intestinal and appetite responses to breakfast porridges from ancient cereal grains:  A MRI pilot study in healthy humans | United Kingdom | To collect initial pilot data on the physiological and GI responses to breakfast porridges made with two millet varieties and oats and rye grains. | Randomised four-way crossover | N | **Participants:**  n = 16 (m = 6, f = 10)  **Age:**  x̄ 20.9 yrs (SD 0.9)  **BMI:**  x̄ 22.1 kg/m^2^ (SD 2.9)  **Condition:**  NIL | **Inclusion:**  - 18–65 years  - Healthy  BMI ≥18 and ≤24·9 kg/m2  - Can give written informed consent  **Exclusion:**  - Using medication which disrupts study measurements  - Participation in another nutritional trial 3 months prior  - Not habitually consuming  3 meals/day  - Not willing to consume study foods  - Working night shifts (between 12:00 and 06:00 hours)  - Partaking in strenuous exercise for >10h/week  - Consumption of 21 alcoholic beverages in a typical week  - Adhering to a medically or self-prescribed diet 2 weeks prior to and during the study  - MRI contraindication  - Pregnancy or inability to lie flat  - Exceeding the scanner bed weight limit (120 kg) | ≥ 10 | 1 week | 5 / 2 | 4 | - 1 week wash out period  - ≥ 10 hr fast  - MRI conducted at baseline to verify stomach contents  - Habitual intake between tests  - Test meal to be consumed within ≤ 15 mins  - Completion of study eligibility check questionnaire  - New VAS sheet provided at each measurement point | **Food:**  Rye Porridge (RP) **(Control)** vs. Pearl Millet Porridge (PMP), Scottish Oats Porridge (SOP), Finger Millet Porridge (FMP)  **Amount:**  RP: 297g, 920kJ, 4.2g P, 49.3g CHO, 2.7g F  PMP: 311g, 920 kJ, 5.3g P, 45.6g CHO, 3.1g F  SOP: 400g, 920 kJ, 7.2g P, 42g CHO, 4.4g F  FMP: 432g, 920 kJ, 3.5g P, 53.1g CHO, 1.3g F  **Fibre dose:**  RP: 6.5g TF  PMP: 6.8g TF  SOP: 8.0g TF  FMP: 13.8g TF | n = 7 (incl. in analysis)  Hunger, DTE and PFC ↓ post breakfast (returned to baseline after 2 hrs). Fullness and satisfaction initially ↓ and then ↑ again in all instances.  AUC hunger (subgroup analysis) indicated a sig. difference between test meals (p = 0.01). Hunger was lower for  PMP compared to oats and rye (n = 10, p = 0.01).  The average appetite score was the lowest post consumption of PMP, but the AUC for this score was not sig. different between all porridges. Millet varieties of the porridge had lower mean scores for hunger, DTE and PFC and higher satisfaction and fullness scores compared to the rye and oat porridges. | - Blood glucose  - Gastric volumes  - Small bowel water content  - Food intake record (post intervention) | Ad libitum meal not provided. Participants free to consume what they desire post intervention (>13:00). | 1 | NIL |
| **Aoe et al., 2014**  **(+)** | Effect of Cooked White Rice with High β-glucan Barley on Appetite and EI in Healthy Japanese Subjects: A Randomized Controlled Trial | Japan | To investigate the effect of cooked white rice containing high β-glucan barley (BAR) on appetite and energy intake. | Randomised crossover | N | **Participants:**  n = 22 (Females)  **Age:**  x̄ 41.9±4.6y  (30-49yrs)  **BMI:**  x̄ 23.3±0.7 kg/m^2^  (22-25 kg/m^2^)  **Condition:**  NIL | **Inclusion:**  **-** Females 30-49yrs  - BMI >22 and <25kg/m^2^  - Consuming 3 meals/day  **Exclusion:**  - Smokers  - Adhering to a diet for the purpose of weight gain/loss  - Partaking in athletic training  - Pregnant or breastfeeding  - Taking medications which could influence appetite  - Allergies or aversion to foods in test meals  - Disordered eaters | ≥ 12 | 1 week | 8 / 4 | 2 | - 1 week wash out period  - ≥ 12 hr fast  - Standardised dinner  - Test meal to be consumed within ≤ 15 mins  - Meal consumed in personal cubicle to prevent effects of social interaction on appetite  - Participants remained in same location for duration of test day  - Participants required to be sedentary between meals | **Food:** White Rice (WR) **(Control)** vs. β-glucan Enriched Barley (BAR)  **Amount:**  WR: 147g, 879kJ, 3.4g P, 48.1g CHO, 0.4g F BAR: 150g, 879kJ, 5.1g P, 42.9g CHO, 0.9g F  **Fibre dose:**  WR: 0.0g TF, 0.0g βG BAR: 5.3g TF, 2.9g βG | n = 21 (incl. in analysis)  Hunger sig. ↓ after BAR consumption compared to WR @240mins (p= 0.032)  Fullness sig. ↑ after BAR consumption compared to WR @240mins (p= 0.038)  Satiety sig. ↑ after BAR consumption compared to WR @60mins (p=0.041) and 480 mins (p=0.027)  Prospective food consumption sig. ↓ after BAR consumption compared to WR @0, 180 & 240mins (p= 0.042, 0.013 & 0.019 respectively) | Energy intake @ lunch sig. ↓ after BAR consumption (3,061 ± 681 kJ) compared to WR (3,280 ± 617 kJ) (p = 0.035).  EI @ dinner did not differ sig. post breakfast interventions. | Lunch options:  - Vegetable sandwich  - Roast beef sandwich  - Spaghetti with ham and mushroom  - Spaghetti with tomato sauce  - Baked fish and scallop  - Roast pork with demi-glaze sauce  Dinner options:  - Bonito carpaccio  - Steamed vegetable  - Spaghetti with spinach and bacon  - Spaghetti Bolognese  - Saute Marlin  - Sauteed chicken with wine vinegar sauce | 1 | NIL |
| **Barone-Lumaga et al., 2012**  **(+)** | Sugar and dietary fibre composition influence, by different hormonal response,  the satiating capacity of a fruit-based and a b-glucan-enriched beverage | Italy | To investigate the satiating effect of three beverages containing different sugar and dietary fibre composition  Monitor the appetite perceptions and blood profile secretion of six GI hormones in the short term as well as overall 24h EI following each beverage. | Randomised crossover | Y | **Participants:**  n = 14 (m = 8, f = 6)  **Age:**  x̄ 27.8±4.9y  (24-39yrs)  **BMI:**  x̄ 20.2±24.6 kg/m^2^  **Condition:**  NIL | **Inclusion:**  - Within the ‘healthy’ BMI range  - Healthy  - Regular breakfast consumer  **Exclusion:**  - Taking medication or drug therapy  - Restrictive/disordered eaters | ≥ 10.5 | 1 week | 4 / 3 | 6 | - 1 week wash out period  - ≥ 10.5 hr fast  - Avoid strenuous physical activity  - Participants isolated during meal time  - Test meal to be consumed within ≤ 15 mins.  - Standardised dinner  - Menstrual cycle phase  - Participants screened for physical/psychological discomfort | **Food:**  Fruit-flavoured Beverage **(Control)** vs. β-glucan Enriched Beverage (BG)  **Amount:**  C: 250mL, 624kJ, 37.3g CHO  BG: 250ml, 617kJ, 0.2g P, 34.5g CHO, 0.3g F  **Fibre dose:**  C: 0.0g βG  BG: 3.0g βG | n =14 (incl. in analysis)  All test beverages vs. sig. ↓ hunger @15, 30, 60mins (p = <0.05) compared to baseline.  BG sig.↑ AUC fullness @60-180mins & 0-180mins (p = <0.05) compared to control.  BG sig. ↑ AUC satiety @60-180mins & 0-180mins (p = <0.05) compared to control. | BG sig. ↓ EI with an 18% decrease at lunch and 40% reduction over 24hrs compared to control. | Selection of Italian style lunches:  - Pasta with tomato sauce  - Pasta with zucchini  - Meat with tomato  - Fish, green salad, chips, bread and fruits. | 0 | NIL |
| **Beck et al., 2009**  **(+)** | Oat β-glucan increases postprandial cholecystokinin levels, decreases insulin response and extends subjective satiety in overweight subjects | Australia | To investigate the effect of β-glucan in extruded breakfast cereals on acute satiety, the dose responsive-ness of such effects, and whether differing processing methods of β-glucan modulates acute satiety responses. | Randomised crossover | N | **Participants:**  n = 14 (m = 7, f = 7)  **Age:**  x̄ 38.7 yrs  (29-45yrs)  **BMI:**  x̄ 29.6 kg/m^2^  (25.2-36.6 kg/m^2^)  **Condition:**  Overweight/obesity | **Inclusion:**  - Aged between 19-45 yrs  - BMI 25 – 36kg/m2  **Exclusion:**  - Smokers  - Disordered eaters  - Allergies/ intolerances | ≥ 10 | 3 days | 4 | 5 | - 3 day wash out period  - ≥ 10 hr fast  - Menstrual cycle phase  - 24 hr dietary recalls collected prior to test day  - Similar nutritional composition of test meals  - Test meal to be consumed within ≤ 10 mins | **Food:** Corn Flakes Cereal **(Control)** vs. Low Dose β-glucan Cereal (LBG), Mid dose β-glucan Cereal (MBG), High dose β-glucan Cereal (HBGO), High Dose β-glucan + Extracted β-glucan Cereal (HBGX),  **Amount:**  C: 39g, 1080kJ, 13.3g P, 43.6g CHO, 3.2g F  LBG: 45g serve, 1098kJ 13.4g P, 43.2g CHO, 3.6g F  MBG: 45g serve, 1106kJ, 13.4g P, 42.9g CHO, 3.8g F  HBGO: 45g serve, 1115kJ, 13.5g P, 42.6g CHO, 4.0g F  HBGX: 45g serve, 1157kJ, 13.0g P, 43.3g CHO, 3.6g F  **Fibre dose:**  C: 1.2g TF, 0g βG  LBG: 3.7g TF, 2.2g βG  MBG: 6.7g TF, 3.8g βG  HBGO: 9.7g TF, 5.5g βG HBGX: 7.8 TF, 5.7g βG | n =14 (incl. in analysis)  All doses of BG indicated a sig. response for fullness (p = 0.017)  Hunger, satiety and prospective food intake neared the level of sig. at 0.05 (p = 0.071, 0.101 and 0.099 respectively).  There were variations in responses to feelings of fullness between HBGO and HBGX. HBGO appeared to enhance feelings of fullness among subjects (although not sig. different, p = 0.013). Data indicated a similar trend of increased satiety with HBGO however not statistically sig.  LBG ↑ satiety (p = 0.039).  All doses demonstrated a sig. effect for ↓ hunger and ↑ satiety (p = 0.039)  No differences in effect were observed between fibres LBG, MBG and HBGO. | Second meal intake:  Energy difference between C and HBGO (>5g BG) was 460kJ.  Sig. difference between C and HBGX groups' lunch (p= 0.033).  No relationship between fibre and gender identified.  - Blood glucose  - Insulin  - CCK  - Ghrelin | Buffet lunch options:  - Assorted sandwiches  - Dried fruit  nuts  - Yoghurt  - Juice | 3 | NIL |
| **Belobrajdic et al., 2019**  **(+)** | High-amylose wheat lowers the postprandial glycaemic response to bread in healthy adults: A randomized controlled crossover trial | Australia | To determine the effects of bread made from high-amylose wheat (HAW) and enriched in RS on postprandial glycemia compared with bread made from conventional low-amylose wheat (LAW) | Randomised crossover | Y | **Participants:**  n = 19 (m = 5, f = 14)  **Age:**  x̄ 30±3y  **BMI:**  x̄ 23±0.7 kg/m^2^  **Condition:**  NIL | **Inclusion:**  **-** Aged between 18-65 yrs  - BMI 18.5 – <27.5kg/m^2^  - Normoglycaemia (3.5-5.5 mmol/L)  **Exclusion:**  - Diabetes  - Smoker  - Pregnant or lactating  - Bleeding disorders  - Food allergy or intolerance to wheat/starchy foods  - Taking medications which can influence glucose tolerance or gastric emptying  - Individuals deemed by investigator as unwilling or unable to comply with study protocol  - Participation in another study in the last 30 days  - History or presence of GI, renal or hepatic disease  - Night shift workers | 12 | 1 week | 10/3 | 7 | - 1 week wash out period  - 12 hr fast  - 24 hr dietary recalls collected prior to test day  - Sensory characteristics of breads were the same  - Test meal to be consumed within ≤ 15 mins  - Avoid high fibre food consumption evening prior  - Avoid strenuous exercise day prior to and morning of test  - Restrict alcohol consumption day prior  - Maintain regular dietary habits and daily routine for study duration | **Food:** Low-amylose Wheat Refined Bread **(Control)** vs. High-amylose Wheat Refined Bread (HAW-R), Low-amylose Wheat Wholemeal Bread (LAW-W), High-amylose Wheat Wholemeal Bread (HAW-W)  **Amount:**  C: 121g, 1045kJ, 10.8g P, 50.0g CHO, 3.4g F  HAW-R: 121g, 951kJ, 13.1g P, 40.0g CHO, 2.8g F  LAW-W: 121g, 984kJ, 12.1g P, 40.0g CHO, 3.7g F  HAW-W: 121g, 922kJ, 15.2g P, 30.0g CHO, 3.6g F  **Fibre dose:**  C: 3.3g TF  HAW-R: 5.5g TF  LAW-W: 8.2g TF  HAW-W: 10.4g TF | n = 19 (incl. in analysis)  Subjective satiety was similar across all 4 test breads (p >0.05).  Satiety sig. ↑ @ 30 mins post consumption of all 4 test breads compared with control glucose beverage (p <0.05). Satiety sig. ↑ @ 60 mins for LAW-W bread compared with control glucose beverage (p <0.01)  Postprandial subjective craving measures were similar across all 4 test breads (p >0.05). Cravings sig. ↓ @ 30 mins post consumption of the wholemeal varieties compared to control glucose beverage (all p <0.05). | - BGLs  - TC  - TGs  - HDL and LDL cholesterol  - Non-esterified free fatty acids  - Insulin  - GIP  - GLP-1  - Ghrelin  - PYY  - Nitrotyrosine  - ICAM-1  - Upper-GI transit rate |  | 1 | NIL |
| **Berti et al., 2015**  **(ø)** | Benefits of breakfast meals and consump-tion pattern on satiety-related sensations in women | Italy | To measure the effect of isoenergetic breakfasts varying in cereal-based food types on satiety-related sensations in adults | Randomised crossover | Y | **Participants:**  n = 9 (Females)  **Age:**  x̄ 25±2.5y  **BMI:**  x̄ 20.5±1.8 kg/m^2^  **Condition:**  NIL | **Inclusion:**  - Female  - Regular breakfast consumer  - No aversions to study foods  **Exclusion:**  - Adhering to a specific diet  - Restrained eaters  - Taking medications | 10.5 | 1 week | 3.5 | 11 | - 1 week wash out period  - 10.5 hr fast  - Participants trained to use VAS | **Food:**  Corn Flakes Cereal (CF) **(Control)** vs. Whole Wheat Flakes (WWF)  **Amount:**  CF: 55g, 1359kJ, 11.5g P, 62.2g CHO, 3.7g F  WWF: 60g, 1339kJ, 13.7g P, 55.6g CHO, 4.8g F  **Fibre dose:**  CF: 1.4g TF  WWF: 9.0g TF | n = 9 (incl. in analysis)  No sig. differences among cereal-based breakfast types on satiety and DTE sensations.  Most breakfasts resulted in sig. ↑ satiety ratings at midday compared to basal values (at 8.30am).  WWF effects on satiety and DTE did not sig. differ from other cereal-based breakfast types despite containing the highest amount of fibre. | Perceived sensations (sensorial characteristics) and breakfast nutritional composition. |  | 0 | NIL |
| **Bodinham, Frost & Robertson et al., 2010**  **(+)** | Acute ingestion of resistant starch reduces food intake in healthy adults | United Kingdom | To investigate the acute effects of consuming 48g of RS type 2 on EI, subjective appetite measures, postprandial glucose and insulin compared to a carbo-hydrate matched placebo supplement. | Randomised crossover | Y | **Participants:**  n = 20 (Males)  **Age:**  x̄ 25.8  (19-31)  **BMI:**  x̄ 23.2±0.65 kg/m^2^  **Condition:**  NIL | **Inclusion:**  - Healthy  - Aged between 19-31 years  - Weight stable in the 3 months prior  **Exclusion:**  - Presence of GI or endocrine disorders  - Restrained eaters | 12 | 1 week | 7/3 | 2 | - 1 week wash out period  - 12 hr fast  - Standardised dinner  - Avoid alcohol ≥ 24 hrs  - Avoid caffeine ≥ 24 hrs  - Avoid strenuous exercise ≥ 24 hrs  - Participants remained in study setting for entire duration  - Minimise activity during study period  - Participants told leftovers could be taken home to prevent over-consumption | **Food:**  Mousse **(Control)** vs. Resistant Starch Mousse  **Amount:**  C: 1595kJ, 9.6g P, 67.2g CHO, 7.9g F  RS: 1595kJ, 9.6g P, 67.2g CHO, 7.9g F  **Fibre dose:**  C: 0.5g TF  RS: 24g TF | n = 20 (incl. in analysis)  No difference in subjective appetite scores relating to hunger, fullness, PFC, or DTE between RS and control supplement. | Energy and macronutrient intake:  48g RS supplement-ation over two meals sig. ↓ EI @ ad libitum meal compared to the EI observed with the placebo supplement (5241kJ and 5606kJ respectively) (p =0.033).  48g RS supplement-ation sig. ↓ EI over the following 24-hr period compared to placebo supp. (12603kJ and 13949kJ respectively) (p = 0.044).  - Postprandial blood glucose  - Insulin  - C-peptide | Pasta based meal @ 7 hours. | 0 | NIL |
| **Breen et al., 2013**  **(+)** | Glycemic, Insulinemic, and Appetite Responses of Patients With Type 2 Diabetes to Commonly Consumed Breads | Ireland | To examine the effect of commonly consumed breads among adults with T2DM on postprandial blood glucose, insulin and appetite responses. | Randomised crossover | N | **Participants:**  n = 11 (m = 7, f = 4)  **Age:**  x̄ 53.9±5.5y  **BMI:**  x̄ 35.1±7.5 kg/m^2^  **Condition:**  T2DM | **Inclusion:**  - Adults with a non-insulin dependent T2DM diagnosis  - Diabetes management through lifestyle intervention and/or oral hypoglycaemic agents  **Exclusion:**  - History of GI disease or gastric surgery  - Taking medications known to influence appetite or GI function | 12 | 1 week | 4.5 | 4 | - 1 week wash out period  - 12 hr fast  - Avoid alcohol  - Avoid excessive evening meals and consumption of pulses prior to test day  - Test meal to be consumed within 10-15 mins  -Participants could not refer to previous VAS ratings when completing a new score | **Food:**  Wheaten White Bread **(Control)** vs.  Wholemeal Soda (WS), Whole Grain Bread (WG), Pumpernickel Rye Bread (PR)  **Amount:**  C: 124g, 1145KJ, 10.8g P, 53.4g CHO, 1.7g F  WS: 131g, 1233kJ, 9.6g P, 57.5g CHO, 2.2g F  WG: 140g, 1364kJ, 12.9g P, 57.5g CHO, 2.9g F  PR: 196g, 1492kJ, 10.0g P, 69.2g CHO, 3.9g F  **Fibre dose:**  C: 3.4g TF  WS: 7.4g TF  WG: 7.5g TF  PR: 19.2g TF | n = 10 (incl. in analysis)  No sig. differences for iAUC for satisfaction, fullness, hunger or PFC among any of the test breads.  PR sig. ↑ feelings of satisfaction (p = 0.01) and fullness (p = 0.04) @ 210 mins compared to C. | - Postprandial blood glucose  - Insulin |  | 1 | 1 partici-pant discont-inued the study due to worsen-ing glycaemic control requiring oral hypogly-cemic treat-ment |
| **Costabile et al., 2018**  **(+)** | Subjective satiety and plasma PYY concen-tration after wholemeal pasta | Italy | To identify acute strategies for appetite regulation and glucose control improve-ment through the use of different pasta meals | Randomised crossover | N | **Participants:**  n = 14 (m = 7, f = 7)  **Age:**  x̄ 30±2y  (20-50yrs)  **BMI:**  x̄ 22±1 kg/m^2^  (18-25 kg/m^2^)  **Condition:**  NIL | **Inclusion:**  - Healthy  - Aged between 20-35 years  - BMI 18-25 kg/m^2^  **Exclusion:**  - History of chronic illness including diabetes, renal failure (serum creatinine >1.7mg/dL), liver disease, anaemia  - Participation in regular strenuous exercise  - Taking medications known to influence glucose and lipid metabolism | 12 | 1 week | 4 | 2 | - 1 week wash out period  - 12 hr fast  - The day prior participants were to:  consume a low fibre diet (≤8g/4180 kJ)  avoid whole grain cereal products, legumes, certain vegetables (i.e., artichokes)  No more than one serve of fruit per day  Avoid vigorous physical activity  - Fibre intake was assessed on test day through a 24 h dietary recall  - Abstain from drinking water for at least 2 hrs prior to study  - Participants limited to 150mL of water with test meal  - Participants not allowed to consume or drink anything else over the next 4 hrs post test meal | **Food:**  Refined Wheat Pasta (RWP) **(Control)** vs. Wholemeal Pasta (WP)  **Amount:**  RWP: 100g, 1322kJ, 11.0g P, 69.2g CHO, 1.3g F  WP: 117g, 1431kJ, 13.8g P, 69.0g CHO, 2.6g F  **Fibre dose:**  RWP: 3.0g TF  WP: 11.0g TF | n = 14 (incl. in analysis)  DTE sig. ↓ @ 120, 150 and 180 mins post consumption of WP compared to RWP (p=0.002).  Hunger sig. ↓ post consumption of WP compared to RWP (p=0.004).  Satiety was not sig. different after consumption of the WP compared to the RWP. However, it was sig. ↑ from 90 to 120mins after consumption of WP compared to RWP (p<0.05). | Self-reported 8-h EI:  218kJ ↓ after consumption of WP compared to RWP, however this was not statistically sig. (p=0.693)  - Ghrelin  - GLP-1  - PYY  - Blood glucose  - Insulin  - TGs | Participants continued their habitual intake. Self-reported EI - 8-h food diary. | 0 | NIL |
| **Emilien et al., 2017**  **(+)** | Effect of resistant wheat starch on subjective appetite and food intake in healthy adults | United States of America | To investigate the effect of replacing 40% of all-purpose flour with an equal weight of RS wheat flour in muffins on EI,  subjective appetite, biomarkers of appetite and glycaemic response | Randomised crossover | Y | **Participants:**  n = 31 (m = 18, f = 13)  **Age:**  x̄ 23±3y  **BMI:**  x̄ 23.45±2.4kg/m^2^  **Condition:**  NIL | **Inclusion:**  - Aged between 18-35 years  - BMI between 18.5 and 29.9 kg/m2  - Regular consumption of breakfast (defined as >5d/week)  **Exclusion:**  - >3kg weight change in previous 3 months  - Previous or current smoker  - Current acute or chronic illness  - Restrained eaters  - Aversion to any of the test foods | 10 | 1 week | 4 | 2 | - 1 week wash out period  - 10 hr fast  - Avoid alcohol  - Avoid strenuous exercise  - Avoid consumption of caffeinated beverages 12 hrs prior to test  - Test meal to be consumed within ≤ 15 mins | **Food:**  Standard Wheat Flour Muffin (SWF) **(Control)** vs. Resistant Wheat Starch Muffin (RWS)  Served with Tropicana orange juice.  **Amount:**  SWF: 3251kJ, 1.06g P, 122.0g CHO, 24.0g F  RWS: 2900kJ, 16.0g P, 102.0g CHO, 24.0g F  **Fibre dose:**  SWF: 2.0g TF  RWS: 26.0g TF | n = 27 (incl. in analysis)  No sig. differences observed for any measures of subjective appetite (no treatment effect after consumption of RWS muffin). | EI sig. ↓ on RWS muffin test day (p = 0.05)  - Blood glucose  - Insulin  - GLP-1  - PYY  - CCK-8  - Ghrelin | Pasta and tomato sauce w/ shredded parmesan cheese (served 4 hrs post BF meal) | 4 | NIL |
| **Flint et al., 2006**  **(+)** | Glycemic and insulinemic responses as determin-ants of appetite in humans | Denmark | To test whether postprandial appetite responses and subsequent EI are influenced by postprandial glycemic and insulinemic responses post consump-tion of a variety of breakfast meals | Randomised crossover | N | **Participants:**  n = 28 (Males)  **Age:**  x̄ 24.8±0.5y  **BMI:**  x̄ 22.5±0.3 kg/m^2^  **Condition:**  NIL | **Inclusion:**  - Healthy males  - Normal weight range  - Non-smokers  **Exclusion:**  - Elite athletes  - History of metabolic disease | 11 | ≥ 1 week | 3.5/3 | 10 | - ≥ 1 week wash out period  - 11 hr fast  - Standardised dinner and dessert prior to test day  - Avoid physical activity and alcohol consumption 2 days prior to test  - Test meal to be consumed within ≤ 15 mins  - Participants to remain sedentary for duration of test | **Food:**  White Wheat Bread **(Control)** vs. German Bread, (GB), Finnish Bread (FB)  All breads served with a butter and cheese portion.  **Amount:**  C: 194g, 2990kJ, 28.0g P, 50.0g CHO, 42.0g F  GB: 216g, 2843kJ, 25.0g P, 50.0g CHO, 41.0g F  FB: 198g, 2882kJ, 25.0g P, 50.0g CHO, 39.0g F  **Fibre dose:**  C: 5.0g TF  GB: 5.0g TF  FB: 10.0g TF | n = 28 (incl. in analysis)  C resulted in sig. ↓ iAOC hunger (i.e., more hunger) compared to FB and GB.  C resulted in sig. ↓ iAOC prospective consumption (i.e., the subjects could consume more) compared to FB and GB. | Ad libitum EI:  No sig. differences in EI @ lunch between breakfast meals.  - Glucose  - Insulin | Pasta salad (13% P, 50% CHO, 37% F) @ Lunch (3.5hrs after test meal) | 0 | NIL |
| **Forsberg, Aman & Landberg 2014**  **(+)** | Effects of whole grain rye crisp bread for breakfast on appetite and energy intake in a subsequent meal: two randomised controlled trails with different amounts of test foods and breakfast energy content | Sweden | To investigate the acute effects on satiety, hunger and DTE as well as EI after a regular breakfast with isoenergetic rye crisp bread or refined wheat bread. | Randomised crossover | Y | **Participants:**  Study 1: n = 21 (m = 10, f = 11)  Study 2: n = 20 (m = 6, f = 14)  **Age:**  x̄ 39±14y  **BMI:**  x̄ 23±3 kg/m^2^  **Condition:**  NIL | **Inclusion:**  - Healthy adults  - Regularly consume 3 meals/day  - Not adhering to a particular diet  **Exclusion:**  - Aged over 65 years  - BMI <18 or >30  - Smokers  - Restrictive eaters/dieters  - Adhering to a vegetarian/vegan diet  - Pregnant, lactating or wishing to conceive during the study period  - Physiological or psychological disturbances with eating i.e., EDs  GI or other medical conditions likely to impact appetite or food intake (incl. food intolerances or allergies) | 12-13 | 6 days | 4 | 4 | - 6 day wash out period  - 12-13 hr fast  - Double blinding  - Low fibre dinner  - Avoid strenuous exercise (24hrs preceding study occasion)  - Refrain from water consumption min. 2 hrs before study commencement  - Conversations relating to the study or rating comparisons were not permitted  - Consumption of food between main meal times was not permitted  - Test meal to be consumed within ≤ 15 mins | **Food:**  Study 1 & 2:  Refined Wheat Bread (RWB) **(Control)** vs. Whole Grain Rye Crispbread (RB)  **Amount:**  Study 1:  RWB: 108g, 1180kJ, 9.7g P, 50.0g CHO, 3.8g F  RB: 80g, 1188kJ, 7.7g P, 52g CHO, 1.9g F  Study 2:  RWB: 86g, 936kJ, 6.9g P, 40.0g CHO, 3.0g F  RB: 64g, 953kJ, 6.1g P, 42.0g CHO, 1.5g F  **Fibre dose:**  Study 1:  RWB: 3.8g TF  RB: 13.0g TF  Study 2:  RWB: 2.6g TF  RB: 10.0g TF | Study 1: n = 21 (incl. in analysis)  Study 2: n = 20 (incl. in analysis)  Study 1:  RB sig. ↓ hunger compared to RWB (p = <0.0001) (differences in hunger through the comparison of AUC was 24%, p = 0.02)  Sig. difference in satiety between RB and WB (p - 0.03) but not when comparing RB and RWB AUCs (p = 0.571)  RB sig. ↓ DTE compared to RWB (p = <0.001). DTE was 23% lower after RB compared to RWB when comparing AUCs (p = 0.02).  Study 2:  RB sig. ↓ hunger compared to RWB (p = <0.0001).  RB sig. ↑ satiety compared to RWB (p = <0.0001, AUC difference 24%, p<0.0001).  RB sig. ↓ DTE compared to RWB (p = <0.0001). | Study 1:  No sig. differences in EI between RB and RWB after the ad libitum lunch.  Study 2:  RB sig. ↓ ad libitum EI (8% reduction, p = 0.024) compared to RWB. | Swedish dish known as ‘Pyttipanna’- a mixture of pieces of pork, beef, potato and onion, fried in 5 g of grape seed oil. Served 4 hours post BF. | 0 | NIL |
| **Freeland, Anderson & Wolever 2009**  **(+)** | Acute effects of dietary fibre and glycaemic carbohyd-rate on appetite and food intake in healthy males | Canada | To assess the effect of equal portions of insoluble fibre (wheat bran) and glycaemic carbohyd-rate (glucose) on subjective sensations of appetite and food intake over 2 hrs in healthy men | Randomised crossover | N | **Participants:**  n = 16 (Males)  **Age:**  x̄ 24.7±1.28y  **BMI:**  x̄ 21.1±1.28 kg/m^2^  **Condition:**  NIL | **Inclusion:**  - Healthy males  - Aged between 18-35 yrs  - BMI 20-27 kg/m2  - Regular consumption of breakfast  **Exclusion:**  - Diagnosis of diabetes  - Smokers  - Adhering to a particular diet  - Restrained eaters  - Taking medication | 10-12 | ≥ 3 days | 2 | 4 | - ≥ 3 day washout period  - 10-12 hr fast  - Questionnaire on sleep habits and stress factors  - Previous VAS ratings were out of view after each rating  - Subjects remained seated for study duration  - Test meal to be consumed within ≤ 15 mins  - Standardised preloads | **Food:**  Wheatlets Cereal (**Control)** vs. Wheat Bran Cereal (Fiber One) (HF)  **Amount:**  C: 44g, 770kJ, 7.65g P, 31.5g CHO, 3.0g F  HF: 90g, 770kJ, 7.65g P, 31.5g CHO, 3.0g F  **Fibre dose:**  C: 1.0g TF  HF: 41.0g TF | n = 16 (incl. in analysis)  No sig. differences found between treatments @ baseline for hunger and fullness.  Fullness scores were sig. ↑ after HF compared to C @ 15 mins (p = 0.04).  AUCs for hunger ↑ and fullness ↓ over time (P < 0.0001)  AUC Hunger and fullness did not differ between treatments @120mins. | - % Energy compensation  - Palatability  - Physical comfort | Individual pizzas (Pepperoni, Three Cheese, Deluxe and Deli Lovers). Served 2 hrs post intervention. | 0 | NIL |
| **Gentile et al., 2015**  **(+)** | Resistant starch and protein intake enhances fat oxidation and feelings of fullness in lean and overweight/obese women | United States of America | To examine the effects of RS alone and in combination with whey protein supplement-ation on energy expenditure, substrate utilisation, and hunger and satiety sensations. | Randomised crossover | Y | **Participants:**  n = 24 (Females)  **Age:**  x̄ 45.8±2.5y  **BMI:**  Overweight/obese cohort: x̄ 31.9±1.4 kg/m^2^  Lean cohort: x̄ 21.0±0.5 kg/m^2^  **Condition:**  Overweight/obesity | **Inclusion:**  - Healthy females  - Non-smokers  - Weight stable (±2 kg) for min. 6 months prior to study  **Exclusion:**  Pre-existing cardiovascular or metabolic diseases | 12 | ≥ 4 days | 4 / 3.15 | 2 | - ≥ 4 day wash-out period  - 12 hr fast  - Standardised daily menu plan the day prior to test day  - Standardised dinner  - Avoid physical activity, caffeine and alcohol min. 24 hrs before  - Test meal to be consumed within ≤ 12 mins  - Subjects to remain sedentary and in a supine position for the duration of study | **Food:**  Pancake with Waxy Maize Starch (WMS) **(Control)** vs. Pancake with Resistant Starch (RS)  **Amount:**  WMS: 1661kJ, 15.0g P, 73.0g CHO, 5.0g F  RS: 1661kJ, 15.0g P, 73.0g CHO, 5.0g F  **Fibre dose:**  WMS: 0.0g TF  RS: 40.0g HDP from WMS | n = 16 (incl. in analysis)  No difference found between the test meals for circulating hunger and satiety sensations.  RS had no effect on perceived hunger or fullness. | - RMR  - Thermic effect of a meal  CHO and fat oxidation  - Glucose  - Insulin  - GLP-1  - GIP  - PYY |  | 8 | NIL |
| **Gonzalez-Anton et al., 2015**  **(+)** | Glycemic Responses, Appetite Ratings and Gastrointestinal Hormone Responses of Most Common Breads Consumed in Spain. A Randomized Control Trial in Healthy Humans | Spain | Assess the glycaemic index, glycemic load, insulinemic index, appetite ratings and postprandial plasma concentra-tions of gastroint-estinal hormones after the consump-tion of five different most common breads consumed in Spain. | Randomised crossover | Y | **Participants:**  n = 23 (m = 13, f = 10)  **Age:**  x̄ 25±1y  (19-32yrs)  **BMI:**  x̄ 23.3±0.5 kg/m^2^  (19.2-28.5 kg/m^2^)  **Condition:**  NIL | **Inclusion:**  - Healthy adults aged between 18-45 yrs  - Regular bread consumers  **Exclusion:**  - Aged <18 yrs and >45 yrs  - BMI <18 or >29  - Smokers  - Pregnant or breastfeeding  - Unusual fibre intake  - Glucose plasma levels >6.1mmol/L  - Insulin plasma levels >10mU/mL  - Blood pressure >110mmHg  - Taking medication for blood pressure, glucose or lipid metabolism  - Metabolic or GI diseases  - Genetic dislipidaemia  - Nutritional supplementation over the last 3 months  - Restrained/disordered eaters  - Partaking in endurance sports | ≥ 10 | ≥ 1 week | 3 | 5 | - ≥ 1 week wash out period  - ≥ 10 hr fast  - Standardised dinner at the same time  - Not permitted to consume or drink anything other than 0.5L water after dinner  - Avoid alcohol and strenuous exercise 2 days prior to test day  - Test meal to be consumed within ≤ 12 mins  - Not permitted to sleep  - Women on menstruation rescheduled | **Food:**  White Bread **(Control)**  vs. Wholemeal Bread (W)  **Amount:**  C: 95g, 1130kJ, 8.8g P, 52.4g CHO, 1.2g F  W: 134g, 1360kJ, 11.8g P, 37.4g CHO, 1.6g F  **Fibre dose:**  C: 3.9g TF  W: 10.6g TF | n = 22 (incl. in analysis)  Appetite ratings for hunger, satiety/fullness, PFC and AUC composite appetite score were similar post consumption of all test breads. | No differences were found in ad libitum EI for the remainder of the intervention day across all test breads.  - Glycaemic index  - GL  - II  - Ghrelin  - GLP-1  - GIP  - PYY  - PP  - Sensory acceptance | Ad libitum lunch: Bolognese macaroni @ 3 hrs. | 1 | NIL |
| **Hamedani et al., 2009**  **(+)** | Reduced energy intake at breakfast is not compen-sated for at lunch if a high-insoluble-fiber cereal replaces a low-fiber cereal | Canada | To compare the effects of a high-insoluble- fibre cereal with a low-fibre cereal on food intake, subjective measures of appetite, and glucose levels in healthy individuals. | Randomised crossover | N | **Participants:**  n = 32 (m = 16, f = 16)  **Age:**  20-26yrs  **BMI:**  20.5-24.5 kg/m^2^  **Condition:**  NIL | **Inclusion:**  - Healthy adults  - Non-smokers  - Regular breakfast eaters  **Exclusion:**  - Diabetes diagnosis  - Restrained/disordered eaters  - Taking medication (i.e., oral contraceptive)  - Irregular menstruation | 10-12 | 1 week | 4.25 / 3 | 2 | - 1 week wash out period  - 10-12 hr fast  - Standardised dinner  - Adhere to normal lifestyle routine  - Avoid alcohol  - Females studied during follicular phase of menstrual cycle  - Questionnaire administered to assess sleep habits and stress factors  - Baseline glucose measured (if >5.6 mmol/L test day rescheduled)  - Test meal to be consumed within ≤ 10 mins  - Previous VAS ratings were out of view after each rating  - Participants to be seated for duration of intervention | **Food:**  Corn Flakes Cereal **(Control)** vs. Wheat Gran Cereal (HF)  **Amount:**  C: 60g, 911kJ, 4.0g P, 52.0g CHO, 0.0g F  HF: 60.0g, 504kJ, 4.0g P, 50.0g CHO, 2.0g F  **Fibre dose:**  C: 1.5g TF, 0.3g IF, 1.2g SF  HF: 28.0g TF, 26.0g IF, 2.0g SF | n = 32 (incl. in analysis)  HF sig. ↑ fullness immediately post consumption compared to C (p= 0.008).  Average appetite AUC did not differ between HF and C (p = 0.7).  No difference in ratings for DTE, hunger or PFC between HF and C. | EI:  HF and C did not affect amount of food consumed @ 180mins post intervention (p = 0.9)  HF sig. ↓ cumulative EI compared to C (1330 ± 57 and 1422 ± 66 kcal, respectively, p = 0.01)  - Glucose  - Satisfaction, physical comfort and palatability | Pizza meal (~837kJ each) consisting of three varieties: deluxe, pepperoni and 3 cheese @ 180 mins after session commenced. | 0 | NIL |
| **Hartvigsen et al., 2014A**  **(+)** | Effects of concen-trated arabinoxylan and b-glucan compared with refined wheat and whole grain rye on glucose and appetite in subjects with the metabolic syndrome: a randomized study | Denmark | To compare the effects of dietary fibre and whole grain on glucose, hormone and appetite responses in subjects with MetS. | Randomised crossover | Y | **Participants:**  n = 15 (m = 7, f = 8)  **Age:**  x̄ 62.8±4.2y  (52-72yrs)  **BMI:**  x̄ 31.1 kg/m^2^  (26.6-37.5 kg/m^2^)  **Condition:**  MetS | **Inclusion:**  - Diagnosis of MetS  - Non-smokers  **Exclusion:**  - Diabetes diagnosis (<7.0mmol/L and HbA1c <6.5%) | 12 | 1 week | 4.5 | 4 | - 1 week wash out period  - 12 hr fast  - Standardised low fibre diet for the day prior  - Avoid alcohol and strenuous exercise  - Avoid taking medication on study day  - Maintain regular lifestyle throughout study  - Not permitted to discuss VAS ratings  - Test meal to be consumed within ≤ 15 mins | **Food:**  Wheat Bread **(Control)** vs. Arabinoxylan bread (AX), Rye bread with kernels (RK), β-glucan bread (BG)  **Amount:**  C: 107g, 1088kJ, 9.0g P, 50.0g CHO, 2.3g F  AX: 136g, 1276kJ, 19.4g P, 50.0g CHO, 2.6g F  RK: 147g, 1040kJ, 7.3g P, 50.0g CHO, 1.8g F  BG: 133g, 1108kJ, 9.8g P, 50.0g CHO, 2.5g F  **Fibre dose:**  C: 2.9g TF, 1.1g AX, 0.2g βG, 0.4g CE, 0.7g RS  AX: 11.2g TF, 7.1g AX, 0.3g βG, 0.6g CE, 0.7g RS  RK: 12.2g TF, 6.1g AX, 1.5g βG, 1.4g CE, 1.1g RS  BG: 13.4g TF, 2.6g AX, 4.2g βG, 4.2g CE, 1.4g RS | n = 15 (incl. in analysis)  AX, BG and RK bread sig.↑ AUC satiety compared to C (p < 0.001, p = 0.001 and p < 0.001 respectively)  AX, BG and RK bread sig. ↓ AUC hunger compared to C (p < 0.001, p = 0.004 and p = 0.004 respectively)  AX and RK bread sig. ↑ AUC fullness compared to WB (p<0.001 and p = 0.002 respectively).  AX, BG and RK bread sig.↓ AUC prospective consumption compared to WB (p < 0.001, p = 0.004 and p = 0.004 respectively). | No effect identified from various bread types on ad libitum food intake (p = 0.089) or cumulative ad libitum food intake (p = 0.071)  - Glucose  - Insulin  - Ghrelin  - GLP-1 | Pizza meal (953kJ/100g) consisting of tomato sauce, ham and cheese @270 mins after session commenced. | 0 | NIL |
| **Hartvigsen et al., 2014A**  **(+)** | Postprandial effects of test meals including concen-trated arabinoxylan and whole grain rye in subjects with the metabolic syndrome: a randomised study | Denmark | To determine the effect of isolated arabinoxylan alone or in combination with whole grain rye kernels on postprandial glucose, insulin, free fatty acids, GI hormones, short chain fatty acids and appetite sensations in subjects with MetS. | Randomised crossover | Y | **Participants:**  n = 15 (m = 8, f = 7)  **Age:**  x̄ 63.5±5.0y  (52-73yrs)  **BMI:**  x̄ 31.3±2.7 kg/m^2^  (26.2-34.7 kg/m^2^)  **Condition:**  MetS | **Inclusion:**  - Diagnosis of MetS  - Non-smokers  **Exclusion:**  - Diabetes diagnosis (<7.0mmol/L and HbA1c <6.5%) | 12 | 1 week | 6 / 4 | 4 | - 1 week wash out period  - 12 hr fast  - Standardised low fibre diet for the day prior  - Avoid alcohol and strenuous exercise  - Avoid taking medication on study day  - Maintain regular lifestyle habits for duration of study  - Not permitted to discuss VAS ratings  - Test meal to be consumed within ≤ 15 mins | **Food:**  Semolina Porridge **(Control)** vs. Arabinoxylan Semolina Porridge (AX), Arabinoxylan-Rye Kernel Semolina Porridge (AXRK), Rye Kernel Semolina Porridge (RK)  **Amount:**  C: 525g, 1005kJ, 8.1g P, 49.5g CHO, 0.7g F  AX: 601g, 1210kJ, 15.9g P, 50.8g CHO, 2.0g F  AXRK: 452g, 1093kJ, 11.5g P, 49.4g CHO, 1.5g F  RK: 435g, 1001kJ, 7.3g P, 49.2g CHO, 1.1g F  **Fibre dose:**  C: 5.7g TF, 0.9g AX, 1.8g RS  AX: 10.2g TF, 3.5g AX, 0.8g AXOS, 1.1g RS  AXRK: 11.5g TF, 4.4g AX, 0.2g AXOS, 1.2g RS  RK: 12.7g TF, 4.7g AX, 1.4g RS | n = 15 (incl. in analysis)  AXRK porridge ↑ satiety @0-360 mins compared to C porridge however this was not statistically sig.  AXRK porridge sig. ↓ hunger after the second standard meal compared to AX porridge and SE porridge (p = 0.002 and p = 0.008 respectively.  AXRK porridge sig. ↓ hunger for the duration of the study more than C porridge (p = 0.005)  No sig. differences found for fullness or prospective consumption between test porridges. | - Glucose  - Insulin  - Ghrelin  - GLP-1  - FFA  - Breath hydrogen  - SCFA | - | 0 | NIL |
| **Heinonen et al., 2007**  **(+)** | Plasma ghrelin levels after two high-carbohyd-rate meals producing different insulin responses in patients with metabolic syndrome | Finland | To ascertain whether circulating ghrelin is affected differently by two varieties of whole-grain breads known to catalyse low or high insulin responses in obese subjects with MeTs. | Randomised crossover | N | **Participants:**  n = 8 (m = 3, f = 5)  **Age:**  x̄ 55.6±1.8y  **BMI:**  x̄ 33.7±0.7 kg/m^2^  **Condition:**  MetS | **Inclusion:**  Needed to fulfill 3/5 following criteria:  - Waist circumference >102cm (men) or >88cm (women)  - Fasting TGs >1.7mmol/l  - Fasting HDL cholesterol >1.0mmol/L (men) or 1.2 mmol/L (women)  - Elevated fasting BGL between 6.1 - 6.9mmol/L  - BP which is = or > 130/85 mm Hg or use of blood pressure medication  **Exclusion:**  - Diabetes diagnosis  - Taking cholesterol-lowering medication or glucocorticoids | 12 | 1 week | 2 | 2 | - 1 week wash out period  - 12 hr fast  - Maintain regular lifestyle  - Avoid strenuous exercise, over-consumption of food and alcohol two days prior to study days  - Avoid physical exertion and smoking a few hours prior to test | **Food:**  Wheat Bread (WB) **(Control)** vs. Whole Grain Rye Bread (WG)  **Amount:**  WB: 125g, 1260kJ, 13.9g P, 50.0g CHO, 5.1g F  WG: 113g, 1093kJ, 6.9g P, 50.0g CHO, 2.6g F  **Fibre dose:**  WB: 7.0g TF  WG: 10.2g TF | n = 8 (incl. in analysis)  No sig. differences in satiety AUC after WG and WB consumption.  Satiety scores were ↑ after ingestion of WB for the duration of the study.  Hunger sig. ↓ after WG and WB consumption (p <0.05) | - Ghrelin  - Glucose  - Insulin |  | 0 | NIL |
| **Holt, Brand-Miller & Stitt 2001**  **(+)** | The effects of equal-energy portions of different breads on blood glucose levels, feelings of fullness and subsequent food intake | Australia | To determine the effects of comer-cially available breads differing in their nutrient composition, energy density and textural qualities on satiety index scores and subsequent ad libitum EI. | Randomised crossover | N | **Participants:**  n = 10 (m = 3, f = 7)  **Age:**  x̄ 23.5±6.2y  (19-39yrs)  **BMI:**  x̄ 22.1±1.3 kg/m^2^  (20-24 kg/m^2^)  **Condition:**  NIL | **Inclusion:**  - Healthy adults aged between 19-40 years  - BMI 18.5-24.9 (normal weight)  **Exclusion:**  - Smokers  - >40 years  - Considered overweight  - Taking prescription medication  - Illness of any kind  - Food insensitivities  - Restrained/disordered eaters  - Family history of diabetes or obesity  - Women regularly experiencing pre-menstrual effects on appetite and food intake | ≥ 10 | 1 week | 2 | 3 | - 1 week wash out period  - ≥ 10 hr fast  - Standardised dinner  - Maintain usual lifestyle habits  - Avoid alcohol and excessive portion sizes the day prior to study  - Test meal to be consumed within ≤ 12 mins  - VAS ratings collected immediately  - Participants remained sedentary for duration of test | **Food:**  Wonder White Bread **(Control)** vs. Coarse White  Bread (CWB), High Fibre Wheat Bread (HF)  **Amount:**  C: 90.1g, 979kJ, 7.3g P, 42.3g CHO, 2.8g F  CWB: 135.4g, 1019kJ, 11.9g P, 63.7g CHO, 0.4g F  HF: 148.2g, 992kJ, 9.6g P, 76.5g CHO, 1.4g F  **Fibre dose:**  C: 1.8g TF  CWB: 15.9g TF  HF: 33.5g TF | n = 10 (incl. in analysis)  The mean SI scores for HF and CW breads were sig. ↑ compared to C (p <0.01) | Subsequent food intake:  Mean SI scores were negatively correlated with EI @ the test meal (120 mins) (p <0.01) and total day EI (p<0.05).  - Glucose | Buffet consisting of:  Breakfast cereals  Toast  Juice  Milk  Tea  Coffee  Biscuits  At conclusion of session (120 mins) | 0 | NIL |
| **Hughes et al., 2022**  **(+)** | Resistant starch wheat increases PYY and decreases GIP but has no effect on self-reported perceptions of satiety | United States of America | To investigate the effects of resistant starch type 2 (RS2) from wheat on perceived appetite sensations and associated GI hormones | Randomised crossover | Y | **Participants:**  n = 30 (m = 12, f = 18)  **Age:**  x̄ 53.9 y  (40-65yrs)  **BMI:**  x̄ 26.5 kg/m^2^  **Condition:**  NIL | **Inclusion:**  - Healthy adults  - BMI between >18.5 - <39.9kg/m^2^  **Exclusion:**  - Untreated or uncontrolled metabolic diseases, GI disorders, cancer or other chronic disease  - Dietary restrictions which inhibited consumption of intervention foods  - Pregnant or lactating  - Smokers  - Using prescription or over-the-counter medications known to impact weight loss or metabolism | 12 | 2 weeks | 4 / 3.5 | 2 | - 2 week wash out period  - 12 hr fast  - Standardised test meal | **Food:**  Wheat Roll (**Control)** vs. RS Wheat Roll (RS)  Served with egg, cheese and turkey sausage.  **Amount:**  C: 3301kJ, 35.2g P, 88.9g CHO, 32.3g F  RS: 3264kJ, 38.0g P, 79.2g CHO, 33.7g F  **Fibre dose:**  C: 4.7g TF, 2.3g IF, 2.4g SF, 1.8g RS  RS: 19.7g TF, 11.8g IF, 7.8g SF, 9.6g RS | n = 30 (incl. in analysis)  No sig. effects of RS on perceived appetite sensations.  No sig. differences between RS and C for AUC hunger, fullness, DTE and prospective consumption. | - GLP-1  - Leptin  - GIP  - Ghrelin  - PYY  - Faecal bile acid analysis  - GI symptoms | Usual intake for remainder of test day captured. | 7 | NIL |
| **Isaksson et al., 2009**  **(+)** | Effect of rye bread breakfasts on subjective hunger and satiety: a randomized controlled trial | Sweden | To compare the satiating effect of  rye bran and intermed-iate rye fraction providing 5g or 8g of dietary fibre per isoenergetic bread breakfast | Randomised crossover | Y | **Participants:**  Study 1:  n = 16 (m = 2, f = 14)  Study 2:  n = 16 (m = 3, f = 13)  **Age:**  Study 1:  x̄ 35±10y  (24-59yrs)  Study 2:  x̄ 38±12y  (23-60yrs)  **BMI:**  Study 1:  x̄ 22±2.8kg/m^2^  Study 2:  x̄ 23±2.0kg/m^2^  **Condition:**  NIL | **Inclusion:**  - Adults aged between 20-60 years  - BMI 18-27kg/m2  - Regular consumption of main meals  - Fasting BGL 4.0-6.1mmol/L  - Haemoglobin 130-170 g/L (men) and 120-150 g/L (women)  - Alaninamino-transferase 0.15-1.1 μkat/L (men) and 0.15 - 0.75 μkat/L (women)  - TSH 0.34-4.0mlE/L  - Willingness to comply with protocol  **Exclusion:**  - Taking medication known to affect appetite or food intake  - Diagnosis of GI related disorders or eating disorders  - Smokers  - Consumption of >3 cups of coffee/day  - >10% body weight change during 3 months prior to participation screening  - Adherence to a diet i.e., vegan, gluten free, weight loss  - Pregnant or lactating or desire to become pregnant during study period | 12 | 6-8 days | 8 | 4-5 | - 6-8 day wash out period  - 12 hr fast  - Avoid alcohol or strenuous physical activity day prior to test day  - Standardised dinner  - Standardised hot drink at mid meal  - Conversation with others not permitted  - Diary used to track food and beverage consumption and physical activity day prior to test day  - Standardised lunch meal on test day  - Use of compliance form  - Test meal and standardised lunch to be consumed within ≤ 30 mins | **Food:**  Study 1:  Wheat Bread **(Control)** vs. Sifted Rye Flour Bread (SRFB), Intermediate Rye Fraction Bread (IRFB), Rye Bran Bread (RBB),  Study 2:  Wheat Bread **(Control)** vs. Bran Bread with 8g DF (BB8), Bran Bread with 5g DF (BB5), Intermediate Fraction Bread with 8g DF (IFB8), Intermediate Fraction Bread with 5g DF (IFB5)  **Amount:**  C: 98g, 1090kJ, 10.0g P, 44.0g CHO, 4.0g F  Study 1:  SRFB: 100g, 1090kJ, 9.5g P, 45.0g CHO, 4.0g F  IRFB: 120g, 1090kJ, 11.0g P, 42.0g CHO, 4.5g F  RBB: 133g, 1090kJ, 13.5g P, 38.0g CHO, 5.5g F  Study 2:  BB5: 114g, 1090kJ, 11.5g P, 42.0g CHO, 4.5g F  IFB5: 123g, 1090kJ, 11.0g P, 42.0g CHO, 4.5g F  IFB8: 126g, 1090kJ, 11.5g P, 41.0g CHO, 4.5g F  BB8: 121g, 1090kJ, 12.0g P, 40.0g CHO, 5.0g F  **Fibre dose:**  C: 1.5g TF  Study 1:  SRFB: 3.5g TF  IRFB: 6.5g TF  RBB: 14.5g TF  Study 2:  BB5: 6.0g TF  IFB5: 6.0g TF  IFB8: 8.5g TF  BB8: 9.0g TF | n = 16 (incl. in analysis study 1 & 2)  Study 1:  RBB, IRFB and SRFB sig. ↑ satiety and sig. ↓ hunger and DTE compared to C (p<0.05)  Study 2:  No effects on hunger and DTE across any of the test breads.  All test breads (excl. IFB8) sig. ↑ satiety compared to C (p<0.05) | - | - | Study 1: 0  Study 2: 3 | NIL |
| **Isaksson et al., 2011**  **(+)** | Rye kernel breakfast increases satiety in the afternoon - an effect of food structure | Sweden | To investigate if variation in the structure of rye grain affects perceived appetite sensations | Randomised crossover | Y | **Participants:**  n = 24 (m = 2, f = 22)  **Age:**  x̄ 25±8y  (20-55yrs)  **BMI:**  x̄ 22.7±2.6 kg/m^2^  (19.3-28.9kg/m^2^)  **Condition:**  NIL | **Inclusion:**  - Adults aged between 20-60 years  - BMI 18-27kg/m2  - Regular consumption of main meals  - Willingness to comply with protocol  **Exclusion:**  - Taking medication known to affect appetite or food intake  - Diagnosis of GI related disorders or eating disorders  - Smokers  - Consumption of >3 cups of coffee/day  - >10% body weight change during 3 months prior to participation screening  - Adherence to a diet i.e., vegan, gluten free, weight loss  - Pregnant or lactating or desire to become pregnant during study period | 12 | 5-15 days | 8 | 3 | - 5-15 day wash out period  - 12 hr fast  - Avoid alcohol or strenuous physical activity day prior to test day  - Standardised dinner  - Conversation pertaining to study not permitted  - Measurement of movement (step count)  - Bagged lunch provided  - Use of compliance form | **Food:**  Wheat Bread **(Control)** vs. Whole Rye Kernel Bread (WRK), Milled Rye Kernel Bread (MRK)  **Amount:**  C: 144g 950kJ, 6.9g P, 38.0g CHO, 3.7g F  WRK: 156g, 1600kJ, 15.6g P, 60.0g CHO, 6.2g F  MRK: 158g, 1650kJ, 15.8g P, 63.0g CHO, 5.7g F  **Fibre dose:**  C: 5.5g TF, 1.6g AX, 0.2g AG, 0.3g βG, 1.4g CE + RS, 0.5g FRU  WRK: 11.6g TF, 4.4g AX, 0.2g AG, 1.0g βG, 2.6g CE + RS, 1.7g FRU  MRK: 13.0g TF, 4.4g AX, 0.2g AG, 1.0g βG, 3.7g CE + RS, 1.6g FRU | n = 24 (incl. in analysis)  No sig. difference in hunger rates between 8:30am to 12:00pm across all bread varieties.  Satiety scores were higher between 8:30am to 12:00pm after consumption of both rye breads compared to WB.  WRK ↓ DTE compared to WB.  WRK and MRK ↑ satiety compared to C.  No sig. difference in any appetite sensation across the entire day between WRK and MRK. | - | - | 3 | NIL |
| **Johansson et al., 2015**  **(+)** | Effects of Unfermen-ted and Fermented Whole Grain Rye Crisp Breads Served as Part of a Standard-ized Breakfast, on Appetite and Postprandial Glucose and Insulin Responses: A Randomized Cross-over Trial | Sweden | To investigate if whole grain rye crisp bread compared to refined wheat crisp bread produces beneficial effects on appetite and postprandial insulin response. | Randomised crossover | N | **Participants:**  n = 23 (m = 7, f = 16)  **Age:**  x̄ 60.1±12.1y  **BMI:**  x̄ 23.8±3.4kg/m^2^  **Condition:**  NIL | **Inclusion:**  - Aged 18-70 years  - BMI 18.5 or 30kg/m2  - Physical activity <2  - Glucose plasma levels <6.0mmol/L  - Insulin plasma levels <11mE/mL  - Serum TSH <2.5mIE/L  - Plasma LDL <5.3 mmol/L  - Fasting plasma TGs <1.8mmol/L  - Regular consumption of main meals  **Exclusion:**  - Smokers  - Taking medications likely to affect appetite  - Medical conditions involving the GIT  - Gluten intolerance or food allergies  - Physical problems with eating  - Dieting or self-reported weight fluctuations of more than 10% of BW in three months prior to eligibility screening  - Recent participation in diet study  - Pregnancy or lactation or desire to become pregnant during study duration | 12 | ≥ 6 days | 4.5 | 3 | - ≥ 6 day wash out period  - 12 hr fast  - Standardised drink of choice on each test occasion  - Avoid eating, drinking and strenuous exercise - 12 hrs prior to test day  - Avoid alcohol and fibre rich foods a day prior to test day  - Subjects confined to study setting and instructed to remain sedentary  - Subjects not permitted to consume or drink any item not included in the study diet  - Conversation pertaining to food, the study or comparison of VAS ratings not permitted  - Test meal and standardised lunch to be consumed within ≤ 15 mins | **Food:**  Yeast-fermented Refined Crispbread (WCB) **(Control)** vs. Yeast-fermented Whole Grain Crispbread (RCB)  **Amount:**  WCB: 52g, 867kJ, 6.3g P, 34.7g CHO, 4.0g F  RCB: 60g, 867kJ, 5.5g P, 38g CHO, 1.3g F  **Fibre dose:**  WCB: 6.0g TF, 2.5g AX, 0.2g AG, 0.1 βG, 1.4g CE + RS, 0.4g Fructan  RCB: 18.3g TF, 8.6g AX, 0.2g AG, 2.1 βG, 2.5g CE + RS, 2.6g Fructan | n = 23 (incl. in analysis)  Hunger ↓ by 12% for RCB compared to WCB (p<0.05).  Fullness ↑ by 16% for RCB compared to WCB (p<0.001).  No sig. differences found for AUC DTE between treatments. | - | - | 2 | NIL |
| **Karalus et al., 2012**  **(+)** | Fermentable Fibers Do Not Affect Satiety or Food Intake by Women Who Do Not Practice Restrained Eating | United States of America | To test the satiating properties of four isolated fibres added to chocolate crisp bars. | Randomised crossover | N | **Participants:**  n = 22 (Females)  **Age:**  x̄ 25 y  (18-38 yrs)  **BMI:**  x̄ 23.7 kg/m^2^  (18-29 kg/m^2^)  **Condition:**  NIL | **Inclusion:**  - Healthy women  - BMI 18-29kg/m^2^  - Regular menstrual cycle  - Regular breakfast consumption  - Typically consume a low fibre diet  **Exclusion:**  - Smokers  - Restrained eaters  - Pregnant or lactating  - Food allergies  - Taking medications  - Taking fibre supplements or probiotics | 12 | ≥ 1 week | 3 | 5 | - ≥ 1 week wash out period  - 12 hr fast  - Menstrual cycle phase  - Adhere to a low fibre diet, avoid alcohol and strenuous exercise one day prior to test day  - Standardised drink of choice on each test occasion  - Test meal to be consumed within ≤ 10 mins  - Carryover effects balanced using a Williams Latin Square Design | **Food:**  No-added Fibre Bar **(Control)** vs. Soluble Corn Fibre Bar (SCF), Resistant Wheat Starch Bar (RWS)  **Amount:**  C: 83g, 1709kJ, 3.1g P, 58.1g CHO, 18.6g F  SCF: 83g, 1609kJ, 2.9g P, 60.2g CHO, 17g F  RWS: 83g, 1604kJ, 2.8g P. 59.7g CHO, 17.2g F  **Fibre dose:**  C: 2.4g TF  SCF: 11.1g TF  RWS: 11.7g TF | n = 22 (incl. in analysis)  No differences in satiety ratings between fibre bars. | EI:  No differences in EI @ lunch or during 24 hrs following test day found between fibre bars.  - Breath hydrogen and methane  - GI tolerance | Pepperoni French bread pizza @ Lunch (3 hrs post breakfast) | 0 | All bars produced more bloating and flatulence comp-ared to C. |
| **Klosterbuer, Thomas & Slavin 2012**  **(+)** | Resistant Starch and Pullulan Reduce Postprandial Glucose, Insulin, and GLP-1, but Have No Effect on Satiety in Healthy Humans | United States of America | To determine the effects of three novel fibres on satiety responses and serum parameters. | Randomised crossover | Y | **Participants:**  n = 20 (m = 10, f = 10)  **Age:**  x̄ 29±8y  (18-38 yrs)  **BMI:**  x̄ 23±2kg/m^2^  **Condition:**  NIL | **Inclusion:**  - Healthy adults aged between 18-60 yrs  - BMI 18.5-27kg/m2  - English speaking  - Weight stable for 3 months prior to study  - Normal fasting BGL  - Regular breakfast consumption  **Exclusion:**  - Smokers  - Restrained eaters/dieters  - GI conditions  - Taking medications  - Food allergies to test products  - Vegetarians  - Regular consumption of >15g fibre/day  - Pregnant or lactating | 12 | ≥ 3 weeks | 4 | 5 | - ≥ 3 week wash out period  - 12 hr fast  - Standardised dinner  - Avoid alcohol and strenuous exercise one day prior to test  - Menstrual cycle phase  - Adhere to a low fibre diet and avoid fibre supplements one day prior to test  - Test meal to be consumed within ≤ 20 mins | **Food:**  Low Fibre Breakfast Meal (muffin + hot cereal) **(Control)** vs. Soluble Corn Fibre Meal (SCF), Resistant Starch Meal (RS)  **Amount:**  C: 2484kJ, 10.4g P, 104.9g CHO, 12.7g F  SCF: 2592kJ, 10.3g P, 103.9g CHO, 12.6g F  RS: 2475kJ, 10.3g P, 105.8 g CHO, 12.8g F  **Fibre dose:**  C: 2.8g TF  SCF: 27.8g TF  RWS: 27.2g TF | n = 20 (incl. in analysis)  AUC hunger, fullness and satisfaction did not differ across fibre treatments. AUC prospective food consumption did not differ between any of the fibre treatments compared to C. | EI:  EI @ Lunch and for the rest of the day did not differ between fibre treatments.  - Glucose  - Insulin  - GLP-1  - Palatability | Buffet lunch consisting of:  -Sandwiches  - Soup  - Salad  - Fresh fruits - Vegetables  - Dessert  - Beverages | 0 | NIL |
| **Korczak et al., 2014**  **(+)** | Bran fibers and satiety in women who do not exhibit restrained eating | United States of America | To determine differences in satiety outcomes to three tests containing either 10 g oat bran, 10 g barley bran and a low fibre condition consumed at dinner and breakfast. | Randomised crossover | Y | **Participants:**  n = 42 (Females)  **Age:**  x̄ 25±4.7y  (20-39 yrs)  **BMI:**  x̄ 21.5±2.2kg/m^2^  (18-29 kg/m^2^)  **Condition:**  NIL | **Inclusion:**  - Healthy women aged between 18-40 years  - BMI 18-29.9kg/m2  - English speaking  - Regular breakfast consumption  - Weight stable for 3 months prior to study  **Exclusion:**  - Smokers  - Taking medications for metabolic conditions  - Restrained eaters  - Vegetarians  - Food allergies to test products  - Diagnosis of cardiovascular disease, diabetes mellitus, cancer in last 5 years, renal or hepatic disease, recent bacterial infection (<2 weeks), GI conditions  - Recent use of antibiotics (within past 6 months)  - Intentional or unintentional weight loss >5kg in past 3 months  - History of drug or alcohol abuse in past 6 months  - High fibre intake (>3 servings of fibre rich foods/day)  - Recent participation in an intervention study (last 30 days)  - Pregnant or lactating  - Women with an irregular menstrual cycle | ≥ 12 | 1 week | 4 | 3 | - 1 week wash out period  - ≥ 12 hr fast  - Standardised dinner  - Menstrual cycle phase  - Subjects remained sedentary for duration of study | **Food:**  Low Fibre Bar **(Control)** vs. Barley Bar (B), Oat Bran Bar (OB)  **Amount:**  C: 103g, 1915kJ, 11.0g P, 68.0g CHO, 16.0g F  B: 98g, 1932kJ, 10.0g P, 72.0g CHO, 17.0g F  OB: 103g, 1940kJ, 9.0g P, 73.0g CHO, 18.0g F  **Fibre dose:**  C: 3.0g TF  B: 10.0g TF  OB: 10.0g TF | n = 42 (incl. in analysis)  No differences between LF, B or OB on any of the satiety measures (hunger, fullness, satisfaction and prospective food intake). | EI:  No sig. differences between treatments in EI @ ad libitum lunch or over the following 24 hours.  - GI tolerance  - Colonic fermentation  - Palatability | Pizza @ Lunch (4 hours post breakfast) | 0 | NIL |
| **Kristensen et al., 2010**  **(+)** | Wholegrain vs. refined wheat bread and pasta. Effect on postprandial glycemia, appetite, and subsequent ad libitum energy intake in young healthy adults | Denmark | To assess the effect of isoenergetic meals containing wholemeal wheat breads and pasta in comparison to similar refined wheat varieties on postprandial glycaemia, appetite sensations and ad libitum EI. | Randomised crossover | N | **Participants:**  n = 16 (m = 6, f = 10)  **Age:**  x̄ 24.1±3.8y  **BMI:**  x̄ 21.7±2.2kg/m^2^  **Condition:**  NIL | **Inclusion:**  Young adults  **Exclusion:**  - Chronic illnesses i.e., diabetes, hypertension, hyperlipidaemia  - Smokers  - Elite athletes (>10h/week)  - Regular medication use (excl. oral contraceptives)  - Taking dietary supplements  - Food intolerances or dislikes for test foods | - | Max. 2 test days/week | 3 | 4 | - Max. 2 tests/week  - Avoid alcohol and strenuous exercise one day prior to test  - Subjects not permitted to consume other food or drinks during study period  - Conversation pertaining to food, appetite or similar topics not permitted | **Food:**  Refined Wheat Bread **(Control)** vs. Whole Grain Wheat Bread (WWB)  Refined Wheat Pasta **(Control)** vs. Whole Grain Wheat Pasta (WWP)  **Amount:**  C-B: 119g, 2000kJ, 24.1g P, 52.9g CHO, 18.1g F  WWB: 146g, 2000kJ, 23.3g P, 60.8g CHO, 15g F  C-P: 72g, 2000kJ, 25g P, 51.9g CHO, 18.2g F  WWP: 83.6g, 2000kJ, 24.7g P, 56.7g CHO, 16.3g F  **Fibre dose:**  C-B: 3.6g TF  WWB: 11.7g TF  C-P: 2.2g TF  WWP: 5g TF | n = 16 (incl. in analysis)  WWB ↑ satiety and fullness compared to C-B, however this was not sig. (p = 0.078 and p = 0.096 respectively).  WWB resulted in larger AUCs for satiety and fullness compared to C-B (22% p < 0.01 and 39% p < 0.01 respectively). It also resulted in lower AUCs for hunger and prospective consumption (-28% p < 0.01 and -31% p < 0.01). | EI:  No sig. effect on EI found.  - Palatability  - Glycaemic response | Pizza slices with tomato sauce, ham and cheese (1060kJ/100g) served 180 min after test meal. | 4 | NIL |
| **Mohr et al., 2021**  **(+)** | Resistant Starch Combined with Whey Protein Increases Postprandial Metabolism and Lowers Glucose and Insulin Responses in Healthy Adult Men | United States of America | To establish the effects of pancake meals containing non-RS constituents or RS type 4 meals with and without greater protein contents on postprandial thermogenesis, fuel utilisation, satiety scores, and gastro-entero-pancreatic hormones | Randomised crossover | Y | **Participants:**  n = 8 (Males)  **Age:**  x̄ 49±13.61y  **BMI:**  x̄ 28.16±5.0kg/m^2^  **Condition:**  NIL | **Inclusion:**  - Healthy men  - Weight stable (±2 kg) > 6 months prior to study commencement  **Exclusion:**  - Smokers  - Diagnosis of cardiometabolic disease | 12 | ≥ 4 days | 5 hrs 15 minutes / 3.5 | 2 | - ≥ 4 day wash-out period  - 12 hr fast  - Standardised daily menu plan the day prior to test day  - Standardised dinner  - Avoid physical activity, caffeine and alcohol min. - 24 hrs before  - Test meal to be consumed within ≤ 12 mins  - Subjects to remain sedentary and in a supine position for the duration of study | **Food:**  Pancake with Waxy Maize Starch **(Control)** vs. Pancake with Resistant Starch (RS)  **Amount:**  C: 1661kJ, 15.0g P, 73.0g CHO, 5.0g F  RS: 1661kJ, 15.0g P, 73.0g CHO, 5.0g F  **Fibre dose:**  C: 45.0g WMS  RS: 40.0g RS from WMS | n = 8 (incl. in analysis)  All satiety and hunger ratings sig. ↓ @ 60 and 120 min post consumption of each test meal (p< 0.002). | - RMR  - Thermic effect of a meal  - Substrate utilisation  - Respiratory exchange ratio  CHO and fat oxidation  - Glucose  - Insulin  - GLP-1  - GIP  - Ghrelin  - PYY  - Heart rate  - Blood pressure |  | 0 | NIL |
| **Peters et al., 2009**  **(+)** | No effect of added b-glucan or of fructooligo-saccharide on appetite or energy intake | Nether-lands | To determine the effect of isoenergetic meal-replacement bars containing fructooligo-saccharide, b-glucan, or both on appetite sensations and food intake across 2 consecutive days. | Randomised crossover | Y | **Participants:**  n = 21 (m = 5, f = 16)  **Age:**  x̄ 52.8y  (36-60yrs)  **BMI:**  x̄ 25.9kg/m^2^  (21.7 – 30.3kg/m^2^)  **Condition:**  NIL | **Inclusion:**  - Healthy adults aged between 18 and 60 years  - BMI ≥21 and ≤32kg/m^2^  - Normal and low-restraint eaters  **Exclusion:**  - Taking medications  - Tendency towards or diagnosis of an eating disorder | 10 | 1 week | 4 | 2 | - 1 week wash out period  - 10 hr fast  - Standardised dinner  - Avoid alcohol and strenuous exercise day prior to test  - Test meal to be consumed within ≤ 15 mins  - Subjects separated at meal time | **Food:**  Control Bar **(Control)** vs. Barley Bar (B)  **Amount:**  C: 56g, 811kJ, 18.7g P, 18.9g CHO, 5.7g F  B: 57g, 819kJ, 19.1g P, 19.0g CHO, 5.8g F  **Fibre dose:**  C: 2.5g TF  B: 4.1g TF | n = 21 (incl. in analysis)  The addition of b-glucan had no effect on appetite ratings.  No statistically sig. difference in subjective hunger ratings between C and B. | EI:  The supplement-ation of b-glucan did not significantly affect EI.  - Viscosity  - GI disturbances | Meat and potato casserole (mashed potatoes, endive, and meat providing 277 kJ, 3g P, 2g F, and 10 g CHO per 100 g) @ Lunch 4 hrs post intervention. | 3 | NIL |
| **Pletsch et al., 2022**  **(+)** | Matched whole grain wheat and refined wheat milled products do not differ in glycemic response or gastric emptying in a randomized, crossover trial | United States of America | To assess the effect of whole grain wheat compared with refined wheat milled products on postprandial glycaemia, gastric emptying and subjective appetite sensations. | Randomised crossover | Y | **Participants:**  n = 16 (m = 7, f = 9)  **Age:**  x̄ 26.6 ± 4.0y  **BMI:**  x̄ 22.2 ± 1.9kg/m^2^  **Condition:**  NIL | **Inclusion:**  - Healthy adults aged between 18 and 60 years  - BMI 18.5-24.9kg/m^2^  **Exclusion:**  - History of diabetes or GI diseases  - Wheat allergies, gluten intolerance or gluten sensitivity | 10 | 1 week | 5 / 4 | 3 | - 1 week wash out period  - 10 hr fast  - Identification of the interventions was hidden from subjects | **Food:**  Refined Grain Flour Porridge **(Control)** vs. Whole Grain Wheat Flour Porridge (WG-F), Reconstituted Whole Grain Wheat Flour with Fine Bran Porridge (RC-F), Reconstituted Whole Grain Wheat Flour with Coarse Bran (RC-Coarse)  **Amount:**  C: 1033kJ, 10.2g P, 50.1g CHO, 0.7g F  WG-F: 1113kJ, 13.0g P, 50.0g CHO, 1.6g F  RC-F: 1155kJ, 13.7g P, 50.1g CHO, 2.3g F  RC-C: 1155kJ, 13.7g P, 50.1g CHO, 2.3g F  **Fibre dose:**  C: 2.5g TF  WG-F: 9.6g TF  RC-F: 9.6g TF  RC-C: 9.6g TF | n = 16 (incl. in analysis)  No difference in effect between treatments on VAS ratings.  No correlation between whole grain porridges and appetite found. | - Postprandial glycaemic response  - Gastric emptying rate |  | 0 | NIL |
| **Rebello et al., 2013**  **(+)** | Acute effect of oatmeal on subjective measures of appetite and satiety compared to a ready-to-eat breakfast cereal: a randomized crossover trial | United States of America | To compare the satiety effect of oatmeal with a popular ready-to-eat breakfast cereal when either was consumed as a breakfast meal. | Randomised crossover | N | **Participants:**  n = 48 (m = 17, f = 29)  **Age:**  x̄ 34.1 ± 14.3y  **BMI:**  x̄ 26.1 ± 7.2kg/m^2^  **Condition:**  NIL | **Inclusion:**  - Healthy adults  - Weight stable in the last 3 months  **Exclusion:**  - Pregnant or lactating  - Restrained eaters/dieters  - Fasting BGL >126 mg/dL  - Allergy or intolerance to oats or milk | 10 | 1 week | 4 | 2 | - 1 week wash out period  - 10 hr fast  - Avoid strenuous exercise day prior to test  - Supervised meal times  - Phase of menstrual cycle  - Test meal to be consumed within ≤ 20 mins | **Food:**  Honey Nut Cheerios **(Control)** vs. Quaker Oatmeal (IO)  **Amount:**  C: 63g, 1050kJ, 4.5g P, 49.9g CHO, 3.4g F  IO: 66g, 1050kJ, 8.3g P, 45.0g CHO, 5.0g F  **Fibre dose:**  C: 4.5g TF, 1.8g SF, 1.7g βG  IO: 6.6g TF, 3.3g SF, 2.6g βG | n = 46 (incl. in analysis)  IO sig. ↓ hunger compared to the RTEC (p = 0.0009) @ 120 minutes (p = 0.0009), 180 minutes (p < 0.0003), and 240 minutes (p = 0.0036).  IO sig.↑ fullness compared to the RTEC (p = 0.005) @ 120 minutes (p = 0.0408), 180 minutes (p = 0.0061), and 240 minutes (p = 0.0102).  IO sig. ↓ DTE compared to the RTEC (p = 0.0002) @ 120 minutes (p = 0.0168), 180 minutes (p < 0.0001), and 240 minutes (p = 0.0022).  IO sig. ↓ prospective food consumption compared to the RTEC (p = 0.0012) @ 120 minutes (p = 0.0058), 180 minutes (p = 0.006), and 240 minutes (p = 0.0047). | - Viscosity |  | 2 | NIL |
| **Rebello et al., 2014**  **(+)** | The role of meal viscosity and oat β-glucan characteristics in human appetite control: A randomized crossover trial | United States of America | To examine the effect of two types of oatmeal and an oat-based ready-to-eat breakfast cereal on appetite sensations, and assess differences in meal viscosity and β-glucan character-istics among the cereals. | Randomised crossover | N | **Participants:**  n = 48 (m = 20, f = 28)  **Age:**  x̄ 29.8 ± 9.9y  **BMI:**  x̄ 27.1 ± 6.7kg/m^2^  **Condition:**  NIL | **Inclusion:**  - Healthy adults  - Not taking medications (excl. birth control or hormone replacement)  - Willing to use an effective birth control method for the duration of the study (females of child bearing capacity)  **Exclusion:**  - Pregnant or lactating - - Unstable weight in the last 3 months  - Fasting BGL >126 mg/dL  - Restrained eaters/dieters  - Allergy or intolerance to oats or milk | 10 | 3 days | 4 | 3 | - 3 day wash out period  - 10 hr fast  - Phase of menstrual cycle  - Avoid alcohol and strenuous exercise one day prior to test  - Supervised meal times  - Test meal to be consumed within ≤ 20 mins  - Subjects required to remain in dining area and refrain from consuming any other food or drink | **Food:**  Honey Nut Cheerios **(Control)** vs. Quaker Oatmeal (IO), Quaker Old Fashioned Oatmeal  **Amount:**  C: 40g, 630kJ, 2.7g P, 30.0g CHO, 2.1g F  IO: 40g, 630kJ, 5.0g P, 27.0g CHO, 3.0g F  SO: 40g, 630kJ, 5.0g P, 27.0g CHO, 3.0g F  **Fibre dose:**  C: 2.7g TF, 1.1g SF, 0.0g βG  IO: 4.0g TF, 2.0g SF, 1.6g βG  SO: 4.0g TF, 2.0g SF, 1.6g βG | n = 48 (incl. in analysis)  AUC hunger over the 4 hours was not statistically different across all breakfast cereals.  IO sig. ↓ hunger @ 60 mins compared to RTEC (p = 0.01).  IO sig. ↑ fullness over the 4 hour period compared to the RTEC (p = 0.04) and at 60 mins (p < 0.01).  IO sig. ↑ fullness at 60 mins compared to SO (p = 0.04) but this was not significantly different over the 4-hour period.  IO sig. ↓ DTE compared to RTEC over the 4 hour period (p = 0.01), at 60 mins (p < 0.01) and 120 minutes (p = 0.01).  IO and SO sig. ↓ prospective intake compared to RTEC over the 4 hour period (p < 0.01).  IO sig. ↓ prospective intake compared to RTEC at 30 minutes (p = 0.01), 60 minutes (p < 0.01) and 120 minutes (p < 0.01).  SO sig. ↓ prospective intake compared to RTEC at 30 minutes (p = 0.02) and 120 minutes (p = 0.02).  O sig. ↓ prospective intake compared to SO at 60 minutes (p = 0.02). | - Viscosity |  | 10 | NIL |
| **Rebello et al., 2016**  **(+)** | Instant Oatmeal Increases Satiety and Reduces Energy Intake Compared to a Ready-to-Eat Oat- Based Breakfast Cereal: A Randomized Crossover Trial | United States of America | To compare the effect of two oat-based breakfast cereals on subjective appetite sensations and food intake. | Randomised crossover | Y | **Participants:**  n = 48 (m = 29, f = 19)  **Age:**  x̄ 32.5 ± 11.1y  (19-63 yrs)  **BMI:**  x̄ 24.9 ± 5.0kg/m^2^  (16.6-38.7kg/m^2^)  **Condition:**  Overweight/obesity (n = 19) | **Inclusion:**  - Healthy adults  - Weight stable in the last 3 months  **Exclusion:**  - Taking regular medications (excl. birth control or hormone replacement therapy)  - Pregnant or lactating  - Fasting glucose >126 mg/dL  - Dieters/restrained eaters  - Allergy or intolerance to oats or milk | ≥ 10 | 1 week | 4 | 2 | - 1 week wash out period  - ≥ 10 hr fast  - Avoid alcohol and strenuous exercise day prior to test  - Supervised meal times  - Standardised test meal  - Test meal to be consumed within ≤ 20 mins | **Food:**  Honey Nut Cheerios **(Control)** vs. Quaker Oatmeal (IO)  **Amount:**  C: 63g, 1046kJ, 4.5g P, 49.9g CHO, 3.4g F  IO: 66g, 1046kJ, 8.3g P, 45.0g CHO, 5.0g F  **Fibre dose:**  C: 4.5g TF, 1.8g SF, 1.7g βG  IO: 6.6g TF, 3.3g SF, 2.6g βG | n = 48 (incl. in analysis)  IO sig. ↓ hunger compared to the C @ 120 minutes (p = 0.005), 180 minutes (p < 0.001), and 240 minutes (p = 0.012).  IO sig.↑ fullness compared to the C @ 120 minutes (p = 0.019), 180 minutes (p = 0.002), and 240 minutes (p = 0.049).  IO sig. ↓ DTE compared to the C @ 120 minutes (p < 0.001), 180 minutes (p < 0.001), and 240 minutes (p = 0.007).  IO sig. ↓ prospective food consumption compared to the C @ 120 minutes (p < 0.001), 180 minutes (p = 0.004), and 240 minutes (p = 0.002). | EI:  EI @ ad libitum lunch meal was sig. ↓ after IO compared to C (p = 0.012) | Sandwiches with a choice of filling:  - Turkey  - Ham  - Roast beef  - Vegetable patty  and  - Potato crisps  - Cookies  Served @ Lunch (4 hrs post BF meal) | 1 | NIL |
| **Rosen et al., 2009**  **(Ø)** | Endosperm and whole grain rye breads are characterized by low post-prandial insulin response and a beneficial blood glucose profile | Sweden | To explore the mechanism for decreased post-prandial insulin demand with rye products | Randomised crossover | N | **Participants:**  n = 12 (m = 9, f = 3)  **Age:**  x̄ 25.3 ± 0.8y  **BMI:**  x̄ 23.1 ± 0.6kg/m^2^  **Condition:**  NIL | **Inclusion:**  - Healthy adults  - Non-smokers  - BMI 18.5-24.9  - Normal BGL  **Exclusion:**  - Smokers  - Receiving drug therapy | 10 | 1 week | 3 | 8 | - 1 week wash out period  - 10 hr fast  - Standardised dinner  - Avoid alcohol and strenuous exercise day prior to test  - Test meal to be consumed within ≤ 12 mins  - Subjects not permitted to consume any additional water or caffeinated beverages during test | **Food:**  White Wheat Bread **(Control)** vs. Endosperm Rye Bread (ERB), Whole Grain Rye Bread (WGRB), Rye Bran Bread (RBB)  **Amount:**  C: 101g, 6.2g P, 40.0g starch, 1.5g F  ERB: 106g, 5.2g P, 45.0g starch, 1.3g F  WGRB: 123g, 6.5g P, 40.0g starch, 1.9g F  RBB: 141g, 9.7g P, 40.0g starch, 2.6g F  **Fibre dose:**  C: 1.8g TF, 0.8g SF, 1.0g IF  ERB: 6.7g TF, 2.5g SF, 4.2g IF  WGRB: 9.6g TF, 2.8g SF, 6.8g IF  RBB: 12.3g TF, 2.0g SF, 10.3g IF | n = 11 (incl. in analysis)  No statistically sig.  difference found in subjective satiety iAUC for the entire 3 hour period between the test breads and C. | - Glucose  - Insulin  - Ghrelin | - | 1 | NIL |
| **Rosen, Ostman & Bjorck 2011A**  **(+)** | Effects of cereal breakfasts on postprandial glucose, appetite regulation and voluntary energy intake at a subsequent standardized lunch; focusing on rye products | Sweden | To evaluate the mechanism through which rye products may reduce postprandial insulin demand and to explore potential appetite regulating properties | Randomised crossover | N | **Participants:**  n = 10 (m = 5, f = 5)  **Age:**  x̄ 26.0 ± 1.1y  **BMI:**  x̄ 22.6 ± 0.4kg/m^2^  **Condition:**  NIL | **Inclusion:**  - Healthy adults  - Non-smokers  - BMI 18.5-24.9  - Normal BGL  **Exclusion:**  - Smokers  - Receiving drug therapy | 10 | 1 week | 6.5 / 4.5 | 7 | - 1 week wash out period  - 10 hr fast  - Standardised dinner  - Avoid alcohol and strenuous exercise day prior to test  - Test meal to be consumed within ≤ 14 mins  - Maintain regular lifestyle throughout study | **Food:**  White wheat bread **(Control)** vs. Endosperm Rye Bread (ERB), Whole Grain Rye Bread (WGRB)  **Amount:**  C: 124g, 1015kJ, 7.3g P, 50.0g starch,  ERB: 134g, 1033kJ, 6.1g P, 50.0g starch  WGRB: 163g, 1078kJ, 8.3g P, 50.0g starch  **Fibre dose:**  C: 6.0g TF, 1.1g RS, 0.5g SF, 4.4g IF  ERB: 17.3g TF, 1.4g RS, 4.0g SF, 11.9g IF  WGRB: 26.3g TF, 2.5g RS, 4.0g SF, 19.8g IF | n = 9 (incl. in analysis)  All test breads ↑ feelings of fullness.  WGRB ↓ DTE compared to C (AUC 0-60 min)  ERB sig. ↓ DTE @0-60mins (p<0.05) compared to C  ERB & WGRB sig. ↓ hunger @0-60mins (p<0.05) compared to C  ERB & WGRB sig. ↑ fullness @0-60mins (p<0.05) compared to C  No sig. difference between ERB & WGRB for hunger, DTE and fullness. | EI:  No sig. difference in effect found between ERB and WGRB compared to C for EI.  - Glucose  - Insulin  - Ghrelin  - FFA  - Adiponectin  - Breath hydrogen | Meatballs, pasta, ketchup and cucumbers for Lunch served @ 270 min after BF. | 0 | NIL |
| **Rosen, Ostman & Bjorck 2011B**  **(+)** | Postprandial glycemia, insulinemia, and satiety responses in healthy subjects after whole grain rye bread made from different rye varieties. 2 | Sweden | To investigate the effect of five rye grain varieties as well as a commercial blend of rye grown in Sweden on postprandial insulin, glucose, and subjective satiety | Randomised crossover | N | **Participants:**  n = 20 (m = 10, f = 10)  **Age:**  x̄ 26.7 ± 0.9y  (21-37 yrs)  **BMI:**  x̄ 22.2 ± 0.39kg/m^2^  **Condition:**  NIL | **Inclusion:**  - Healthy adults  - Non-smokers  - BMI 18.5-24.9  - Normal BGL  **Exclusion:**  - Smokers  - Receiving drug therapy | 10 | 1 week | 3 | 7 | - 1 week wash out period  - 10 hr fast  - Standardised dinner  - Avoid alcohol and strenuous exercise day prior to test  - Test meal to be consumed within ≤ 14 mins  - Subjects to remain sedentary post test | **Food:**  White wheat bread **(Control)** vs. Amilo (A), Evolo (E), Kaskelott (K), Picasso (Pi), Vicello (V), Wholegrain Rye (WGR)  **Amount:**  C: 125g, 6.7g P, 50.0g starch,  A: 154g, 9.0g P, 50.0g starch  E: 152g, 9.3g P, 50.0g starch  K: 154g, 10.0g P, 50.0g starch  Pi: 153g, 9.7g P, 50.0g starch  V: 148g, 10.1g P, 50.0g starch  WGR: 157g, 10.6g P, 50.0g starch  **Fibre dose:**  C: 2.8g TF, 0.4g SF, 2.4g IF  A: 12.6g TF, 3.6g SF, 9.0g IF  E: 13.3g TF, 4.0g SF, 9.3g IF  K: 13.8g TF, 3.8g SF, 10.0g IF  Pi: 13.4g TF, 3.7g SF, 9.7g IF  V: 13.1g TF, 3.0g SF, 10.1g IF  WGR: 13.9g TF, 3.3g SF, 10.6g IF | n = 15 (incl. in analysis)  WG sig. ↓ hunger and DTE compared to C (p<0.05)  E sig. ↓ hunger @0-60 min and 0-180mins (p<0.05) compared to C  E sig. ↑ fullness @60-120min and sig. ↓ hunger @0-180min compared to C.  V sig. ↑ fullness @0-60min and compared to A (p<0.05).  High IF sig. ↑ satiety @0-60min (p <0.05).  Higher IF sig. ↓ DTE @60-120min (p <0.05) | - Glucose  - Insulin | - | 1 | NIL |
| **Schroeder et al., 2009**  **(+)** | Influence of whole grain barley, whole grain wheat, and refined rice-based foods on short-term satiety and energy intake | United States of America | To compare the effect of whole grain high-fibre barley, whole grain wheat and refined rice-based foods on satiety and EI | Randomised crossover | Y | n = 47 (m = 12, f = 35)  **Age:**  x̄ 31y  (19-58 yrs)  **BMI:**  x̄ 23kg/m^2^  (18.8-30.7kg/m^2^)  **Condition:**  NIL | **Inclusion:**  - Healthy adults aged between 18 and 65  - Regularly consume main meals  - Stable weight  **Exclusion:**  - BMI <18 or >40kg/m^2^  - Smokers  - Athletes  - Pregnant or lactating  - Food allergies  - Dieters or restrained/disordered eaters  - Taking medications known to affect appetite | 10 | 1 week | 4 | 3 | - 1 week wash out period  - 10 hr fast  - Subjects remained in the lab for test duration  - Test meal to be consumed within ≤ 20 mins  - Subjects limited to sedentary activity  - Refined grain product used in ad libitum lunch to prevent confounding between whole grain test foods and non-treatment whole grains | **Food:**  Refined Rice Snack Mix **(Control)** vs. Whole Wheat Snack Mix (WWM), Sustagrain Barley Snack Mix (SBM)  Refined Rice Cereal **(Control)** vs. Whole Wheat Cereal (WWC), Sustagrain Barley Cereal (SBC)  **Amount:**  C-M: 30.0g, 502kJ, 2.0g P, 26.0g CHO, 1.0g F  WWM: 30.0g, 460kJ, 3.0.0g P, 24g CHO, 1g F  SBM: 30.0g, 502kJ, 4.0g P, 23.0g CHO, 2.0g F  C-C: 56g, 879kJ, 3.0g P, 47g CHO, 0.5g F  WWC: 56g, 836kJ, 5.0g P, 44g CHO, 1.0g F  SBC: 56g, 921kJ, 8.0g P, 40.0g CHO, 3.0g F  **Fibre dose:**  C-M: 0.0g TF,  WWM: 2.0g TF, 0.2g SF, 1.8g IF  SBM: 6.0g TF, 2.4g SF, 3.6g IF  C-C: 1.0g TF, 0.1g SF, 0.9g IF  WWC: 5.0g TF, 0.6g SF, 4.4g IF  SBC: 12.0g TF, 4.8g SF, 7.2g IF | n = 47 (incl. in analysis)  No sig. differences in the AUC subjective appetite ratings among the three product types for hunger, fullness, DTE or prospective food consumption.  No sig. differences in appetite scores across the tproduct types for any parameter at each measurement point.  Subjects reported sig. less hunger prior to lunch for barley varieties (p = 0.002) but not for wheat or rice compared to baseline.  Subjects reported feeling sig. fuller at prior to lunch compared to before breakfast for barley (p = 0.001), wheat (p = 0.011), and rice (p = 0.044) tests. | EI:  EI @ ad libitum lunch did not statistically differ across the product types. | Turkey sandwich on refined wheat bread with cheese, mustard, mayonnaise, yoghurt, an apple, a banana, saltine crackers, chicken noodle soup, tomato soup, carrot sticks, cookies, potato chips, Sprite, Coke, Diet Coke and water served @ lunch (3.5 hours post BF) | 3 | NIL |
| **Stefoska-Needham et al., 2016**  **(+)** | Flaked sorghum biscuits increase postprandial GLP-1 and GIP levels and extend subjective satiety in healthy subjects | Australia | To test the effects of three different ready-to-eat whole grain sorghum flaked breakfast biscuits on appetite responses | Randomised crossover | Y | n = 40 (m = 20, f = 20)  **Age:**  M: x̄ 27.7 ± 6.921y  (21-43yrs)  F: 31.0±8.891y  (21-50yrs)  **BMI:**  M: 23.78 ± 2.025kg/m^2^  F: 22.94 ± 3.00kg/m^2^  **Condition:**  NIL | **Inclusion:**  - Healthy adults aged between 18-50 years  - BMI 20-31kg/m^2^  **Exclusion:**  - Smokers  - Restrained eaters  - Diagnosis of a serious illness such as diabetes  - Food allergies  - Pregnant or lactating  - Postmenopausal | 12 | ≥ 3 days | 4 | 4 | - ≥ 3 day washout period  - 12 hr fast  - Standardised dinner  - Avoid alcohol and strenuous exercise one day prior to test  - Phase of menstrual cycle  - Test meal to be consumed within ≤ 10 mins | **Food:**  Wheat Flaked Breakfast Cereal **(Control)** vs. White Sorghum Flaked Breakfast Cereal (WSC), Red Sorghum Flaked Breakfast Cereal (RSC), Brown Sorghum Flaked Breakfast Cereal (BSC)  **Amount:**  C: 50g, 754kJ, 6.9g P, 33.5g CHO, 0.86g F  WSC: 50g, 776kJ, 5.4g P, 35.5g CHO, 1.5g F  RSC: 50g, 775kJ, 4.7g P, 35.9g CHO, 1.5g F  BSC: 50g, 765kJ, 5.9g P, 33.7g CHO, 1.5g F  **Fibre dose:**  C: 4.8g TF  WSC: 3.7g TF  RSC: 3.9g TF  BSC: 4.9g TF | n = 40 (incl. in analysis)  VAS indicated that the treatment effect was sig. in all questions (p < 0.001).  No differences between sorghum biscuits were found.  Means of VAS scores indicated sig. ↑ hunger and ↓ satiety ratings after consumption of control biscuits compared to all three sorghum varieties over the 4 hours. | EI:  No sig. differences in lunch EI for the group or between genders across the test products. No sig. differences in total EI for the remainder of the day or in combination with the ad-libitum lunch.  - Glucose  - Insulin  - Ghrelin  - GIP  - GLP-1  - PYY | Variety of sandwiches, cold pasta salad, dried fruit, yoghurt and water (~7500kJ, containing 50% CHO, 20% P and 30% F) served at Lunch (4 hours post BF). | 0 | NIL |
| **Stewart et al., 2018**  **(+)** | Type-4 Resistant Starch in Substitution for Available Carbo-hydrate Reduces Postprandial Glycemic Response and Hunger in Acute, Randomized, Double-Blind, Controlled Study | United States of America | To assess the postprandial glucose response, postprandial satiety and gastro-intestinal tolerance after consumption of a high fibre scone containing a novel RS4 or a low fibre control scone without RS4 | Randomised crossover | Y | **Participants:**  n = 35 (m = 12, f = 23)  **Age:**  x̄ 46.2 ± 2.2y  **BMI:**  x̄ 26.1 ± 0.5kg/m^2^  **Condition:**  NIL | **Inclusion:**  - Healthy adults aged between 18-74 years  - BMI 18.5-29.99kg/m2  - Willing to commit to a medically approved contraception method for female subjects capable of bearing a child  **Exclusion:**  - Fasting capillary glucose ≥ 5.55 mmol/L  -Significant trauma or surgery within 3 months of eligibility screening  - Recent or current history of drug or alcohol misuse  - Weight change (>4.5kg) in past 2 months prior to eligibility screening  - Unmanaged hypertension  - Recent use of antibiotics  - Symptoms of current infection  - Extreme dietary behaviours  - Recent consumption of foods fortified and/or containing probiotics  - Use of medications known to impact CHO metabolism, GI motility, satiety, appetite, taste, sense of smell, or weight  - Pregnant or lactating | - | 1 week | 3 | 3 | - 1 week washout period  - Avoid changes in smoking habits or nicotine use  - Avoid use of tobacco products during test visits  - Avoid alcohol and vigorous exercise one day before tests  - Subjects agreed to maintaining body weight and habitual diet for duration of study  - Standardised diet one day before each test  - Phase of menstrual cycle  - Test meal to be consumed within ≤ 10 mins | **Food:**  Control Scone **(Control)** vs.  Fibre Scone  **Amount:**  C: 84g, 1372kJ, 7.1g P, 42.8g CHO, 16.0g F  F: 84g, 1130kJ, 6.1g P, 28.9g CHO, 14.4g F  **Fibre dose:**  C: 4.0g TF  F: 14.9g TF | n = 35 (incl. in analysis)  Hunger and DTE AUC 0-180min sig. ↓ after consumption of the fibre scone compared to C (p < 0.05). Fullness and prospective consumption AUC 0-180min did not sig. differ between tests. | - Glucose  - Insulin  - GI tolerability  - Palatability | - | 1 | NIL |
| **Vitaglione et al., 2009**  **(+)** | β-Glucan-enriched bread reduces energy intake and modifies plasma ghrelin and peptide YY concen-trations in the short term | Italy | To evaluate the effect of barley b-glucans on acute appetite and on satiety-related hormones | Randomised crossover | N | **Participants:**  n = 14 (m = 7, f = 7)  **Age:**  x̄ 23.9 ± 3.0y  (20-29 yrs)  **BMI:**  x̄ 22.9 ± 2.8kg/m^2^  **Condition:**  NIL | **Inclusion:**  - Healthy  - Regular breakfast consumption  **Exclusion:**  - Taking medications  - Restrained eaters | ≥ 10.5 | 1 week | 3 | 4 | - 1 week washout period  - ≥ 10.5 hr fast  - Standardised dinner  - Phase of menstrual cycle  - Physical or psychological discomfort  - Avoid physical activity day before test | **Food:**  Wheat Flour Bread **(Control)** vs. β-glucan Enriched Bread  (βG)  **Amount:**  C: 93g, 1084kJ, 8.4g P, 54.8g CHO, 2.1g F  βG: 100g, 1092kJ, 7.7g P, 54.5g CHO, 2.5g F  **Fibre dose:**  C: 1.4g TF, 0.0g βG  βG: 4.4g TF, 3.0g βG | n = 14 (incl. in analysis)  βG sig. ↓ hunger @ 60-180 min (AUC: 49% decrease, p < 0.05) compared to C  βG sig. ↑ fullness @ 60-180min (AUC: 25% increase, p < 0.05) compared to C  βG sig. ↑ satiety @ 60-180 min (AUC: 55% increase, p < 0.05) compared to C | EI:  βG sig. ↓ intake @ Lunch compared to C (AUC: 19% decrease)  - Glucose  - Insulin  - Ghrelin  - PYY | Italian-style lunch consisting of pasta with tomato sauce, cold rice salad, meat, fish, green salad, chips, bread and fruits served 3 hrs post BF. | 0 | NIL |
| **Vuholm et al., 2014**  **(+)** | Appetite and food intake after consumption of sausages with 10% fat and added wheat or rye bran | Denmark | To investigate if appetite sensations and EI was affected by the addition of dietary fibres to sausages | Randomised crossover | Y | **Participants:**  n = 25 (Males)  **Age:**  x̄ 25.2 ± 4.0y  **BMI:**  x̄ 23.2 ± 2.0kg/m^2^  **Condition:**  NIL | **Inclusion:**  - Healthy males aged between 20-40 years  - BMI 19-28kg/m^2^  **Exclusion:**  - Diagnosis of chronic illness  - Taking medication  - Smokers  - Participation in other studies  - Taking dietary supplements  - Allergies, intolerances or dislikes of foods included in study | 12 | ≥ 3 days | 4 | 4 | - ≥ 3 day washout period  - 12 hr fast  - Avoid alcohol and strenuous activity  - Standardised dinner  - Other food or drink not permitted  - Sedentary activity permitted only  - Conversations about food, appetite or related topics not permitted  - Subjects monitored during test day  - Test meal to be consumed within ≤ 10 mins | **Food:**  Wheat Flour Sausage Meal **(Control)** vs. Wheat Bran Sausage Meal (WB), Rye Bran Sausage Meal (RB)  **Amount:**  C: 373g, 2753kJ, 31.6g P, 82.6g CHO, 21.4g F  WB: 382g, 2902kJ, 35.2g P, 84.1g CHO, 23g F  RB: 388g, 2875kJ, 34.5g P, 84.2g CHO, 22.5g F  **Fibre dose:**  C: 4.4g TF  WB: 10.4g TF  RB: 9.6g TF | n = 25 (incl. in analysis)  Overall sig. positive effect of sausage meal on all four appetite sensations including satiety (p = 0.001), hunger (p < 0.001), prospective consumption (p < 0.001) and fullness (p < 0.003).  RB and WB sig. ↓ hunger compared to WF (p < 0.001).  RB and WB sig. ↑ satiety compared to WF (p<0.001 and p=0.005 respectively).  RB and WB sig. ↑ fullness compared to WF (p = 0.001 and p = 0.012 respectively).  Effect of RB compared to WB did not differ. Overall satiety was not affected by type of meal.  RB sig. ↑ satiety, ↓ hunger and ↓ intake compared to WF (iAUC p < 0.001)  RB sig. ↑ fullness compared to WF (iAUC p < 0.01)  WB sig. ↑ fullness compared to WF (iAUC p < 0.05)  WB sig. ↑ satiety compared to WF (iAUC: p < 0.01)  WB sig. ↓ hunger and intake compared to WF (iAUC: p < 0.001)  RB and WB sig. ↓ prospective EI (p < 0.001)  RB sig. ↓ prospective EI compared to WF (p = 0.003) | EI:  Ad libitum EI did not differ between the all test meals.  - GI discomfort  - Palatability | Pasta Bolognese served at Lunch with an energy content of 556 kJ/100 g (15.2 E% protein, 30.0 E% fat and 54.8 E% CHO) | 3 | NIL |
| **Weickert et al., 2006**  **(Ø)** | Wheat-fibre-induced changes of postprandial peptide YY and ghrelin responses are not associated with acute alterations of satiety | Germany | To investigate effects of purified insoluble cereal fibres on postprandial PYY and ghrelin responses as well as satiety ratings | Randomised crossover | Y | **Participants:**  n = 14 (Females)  **Age:**  x̄ 23.6 ± 1.69y  **BMI:**  x̄ 21.3 ± 1.5kg/m^2^  **Condition:**  NIL | **Inclusion:**  - Healthy women  - No menstrual irregularities  **Exclusion:**  - Pregnancy | 10 | 1 week | 5 | 6 | - 1 week wash out period  - 10 hr fast  - Avoid strenuous activity 3 days prior to test  - Subjects to comply with standardised diet day prior to intervention | **Food:**  White Bread **(Control)** vs. Wheat Fibre Bread (WF), Oat Fibre Bread (OF)  **Amount:**  C: 103g, 1800kJ, 7.2g P, 50.0g CHO, 0.82g F  WF: 131g, 1012kJ, 7.3g P, 50.0g CHO, 0.85g F  OF: 133g, 1800kJ, 7.3g P, 50.0g CHO, 0.84g F  **Fibre dose:**  C: 2.9g TF  WF: 10.5g TF  OF: 10.6g TF | n = 14 (incl. in analysis)  Hunger sig. ↓ after consumption of all test meals @ 30 mins (p < 0.0001).  No difference between hunger scores for each test meal. | - Ghrelin  - PYY | - | 0 | NIL |
| **Willis et al., 2009**  **(+)** | Greater satiety response with resistant starch and corn bran in human subjects | United States of America | To compare a low fibre muffin with a variety of high fibre muffins containing different fibre types on their effects on satiety | Randomised crossover | Y | **Participants:**  n = 20 (m = 7, f = 13)  **Age:**  x̄ 28.4 ± 4.0y  (20-54yrs)  **BMI:**  x̄ 22.9 ± 0.6kg/m^2^  (19.7-26.9 kg/m^2^)  **Condition:**  NIL | **Inclusion:**  - Healthy adults aged between 18-65 years  - English speaking  - BMI <30kg/m2  **Exclusion:**  - Smoker  - History of disease or GI issues  - Taking medications  - Pregnant or lactating  - Recent weight change | 12 | 1 week | 3 | 5 | - 1 week wash out period  - 12 hr fast  - Avoid strenuous exercise/that may alter body weight  - Maintain regular eating habits  - Phase of menstrual cycle  - Test meal to be consumed within ≤ 10 mins  - Subjects separated during meal time and remained sedentary for duration of study | **Food:**  Low Fibre Muffin **(Control)** vs. Resistant Starch Muffin (RS), Barley β-glucan + oat fibre muffin (βG), Corn Bran Muffin (CB)  **Amount:**  C: 76g, 745kJ, 3.9g P, 2.9g F  RS: 92g, 729kJ, 3.8g P, 3.3g F  βG: 96g, 733kJ, 3.5g P, 3g F  CB: 99g, 729kJ, 4g P, 3.5g F  **Fibre dose:**  C: 1.6g TF, 0.4g SF, 1.1g IF  RS: 8.0g TF, 0.1g SF, 7.9g IF  βG: 9.4g TF, 4.0g SF, 5.3g IF  CB: 9.6g TF, 0.0g SF, 9.6g IF | n = 20 (incl. in analysis)  CB ↓ hunger compared to C (AUC p = 0.056)  CB sig. ↓ prospective consumption compared to C (AUC p = 0.025)  RS sig. ↓ hunger and appetite compared to C (AUC p = 0.06, 0.009)  CB sig. ↑ fullness and satiety compared to PD and C (p < 0.049)  RS sig. ↓ hunger from 0-120 mins compared to C.  RS sig. ↑ fullness from 0-180mins compared to C.  CB sig. ↓ hunger @ 60mins, ↑ satiety @ 120mins and ↑ fullness @ 180mins compared to C.  RS and CB sig.↓ prospective food consumption compared to C | Characteristics of palatability i.e., visual appeal, smell taste and overall pleasantness | - | 0 | NIL |
| **Wolever et al., 2020**  **(+)** | Increasing oat β-glucan viscosity in a breakfast meal slows gastric emptying and reduces glycemic and insulinemic responses but has no effect on appetite, food intake, or plasma ghrelin and PYY responses in healthy humans: A randomized, placebo-controlled, crossover trial | Canada | To determine the effect of altering the amount or MW and viscosity of oat b-glucan in a breakfast meal on the primary endpoint of food intake at a subsequent meal. | Randomised crossover | Y | **Participants:**  n = 28 (m = 14, f = 14)  **Age:**  x̄ 33.1 ± 10.6y  **BMI:**  x̄ 24.8 ± 2.3kg/m^2^  **Condition:**  Overweight (n = 14) | **Inclusion:**  - Healthy adults aged between 18-60 years  - Fasting serum glucose <7.0mmol/L  - BMI ≥ 20.0 and ≤ 30.0 kg/m^2^  **Exclusion:**  - Smokers  - Restrained eaters  - Pregnant or lactating  - No prior participation in food intake studies | - | ≥ 5 days | 4.5 / 3 | 4 | - ≥ 5 days wash out period  - Standardised dinner prior to test  - Subjects to maintain lifestyle habits for duration of study  - Avoid alcohol or physical activity one day prior  - Standardised ad libitum meal  - Test meal to be consumed within ≤ 15 mins | **Food:**  Cream of Rice **(Control)** vs. Oatmeal plus 3.0g Oat-Bran (OBG2g), Oatmeal plus 10.1g Oat-Bran (OBG4g)  **Amount:**  C: 325g, 1582kJ, 15.8g P, 52.7g CHO, 12.2g F  OBG2g: 332g, 1573kJ, 16.0g P, 56.8g CHO, 11.9g F  OBG4g: 336g, 1582kJ, 16.0g P, 60.4g CHO, 12.2g F  **Fibre dose:**  C: 1.4g TF, 0.0g βG  OBG2g: 5.6g TF, 2.0g βG  OBG4g: 9.3g TF, 4.0g βG | n = 28 (incl. in analysis)  No sig. difference between CR and any of the oatmeal tests for total AUC for any measure of subjective appetite.  Over the 3-hr period OBG2g appeared the least satiating of the test meals however no sig. difference found between CR and any of the other tests at any point. | EI:  No sig. difference in EI between CR and any of the oatmeal tests. | Individual pizza meal consisting of Pepperoni or Three Cheese served @ Lunch (3 hrs post intervention) | 5 | NIL |
| **Ye et al., 2015**  **(+)** | Soluble dietary fiber (Fibersol-2) decreased hunger and increased satiety hormones in humans when ingested with a meal | United States of America | To assess the effect of Fibersol-2, a water-soluble, nonviscous and highly digestion-resistant maltodextrin on hunger and satiety as well as gut satiety factors in healthy humans. | Randomised crossover | Y | **Participants:**  n = 19 (m = 9, f = 10)  **Age:**  x̄ 36.0 ± 16.0y  (20-62 yrs)  **BMI:**  x̄ 25.0 ± 2.0kg/m^2^  (21.1-28.2 kg/m^2^)  **Condition:**  NIL | **Inclusion:**  - Healthy adults aged between 20-65 years  - BMI ≥ 21 and ≤ 28 kg/m^2^  **Exclusion:**  - Smokers  - Taking medication  - Restrained eaters  - Dislike for the test meal | 13 | 1 week | 4 | 3 | - 1 week wash out period  - 13 hr fast  - Standardised dinner  - Avoid alcohol one day prior to test  - Test meal to be consumed within ≤ 15 mins | **Food:**  Control Tea **(Control)** vs. Tea with 5g Fibersol-2 (FT5g), Tea with 10g Fibersol-2 (FT10g)  **Fibre dose:**  C: 0.0g TF  FT5g: 5.0g TF  FT10g: 10.0g TF | n = 19 (incl. in analysis)  FT10 delayed hunger from first post meal assessment.  FT10 prolonged satiety compared to C and FT5.  FT10 resulted in feelings of fullness for 1.5 hours post intervention, compared to C and FT5. C and FT5 did not diminish hunger increase post intervention and hunger and satiety ratings differed significantly at all measurement points proportional to the initial post intervention assessment (p < 0.05).  FT10 sig. ↓ AUC hunger and ↑ AUC satiety compared to C (p < 0.05) | - Ghrelin  - CCK  - Gastrin  - GIP  - PYY  - GLP-1  - Palatability  - GI symptoms | - | 1 | NIL |
| **Zamarat-skaia et al., 2017**  **(+)** | Impact of sourdough fermentation on appetite and postprandial metabolic responses-A randomised cross-over trial with whole grain rye crispbread | Sweden | To determine the effect of consuming sourdough-fermented and unfermented rye crispbread on self-rated appetite, postprandial glucose and insulin response in healthy subjects. | Randomised crossover | Y | **Participants:**  n = 24 (m = 13, f = 11)  **Age:**  x̄ 30.0 ± 11.0y  **BMI:**  x̄ 23.0 ± 5.0kg/m^2^  **Condition:**  NIL | **Inclusion:**  - Healthy adults aged between 18-70 years  - BMI 18.5-30kg/m^2^  - Physical activity level <2  - Fasting plasma glucose <6·0 mmol/l  - Fasting serum insulin <11 mE/l  - Serum thyroid-stimulating hormone <2·5 mIE/l  - Plasma LDL <5·3 mmol/l  - Fasting plasma TAG <1·8 mmol/l  - Regular consumption of main meals  - Weight stable  **Exclusion:**  - Smokers  - Taking medication with appetite effects  - Diagnosis of GI disorders  - Food allergies or gluten intolerance  - Physical problems with eating  - Dieters  - Recent participation in dietary study  - Pregnant or lactating | 10 | ≥ 6 days | 7 | 3 | - ≥ 6 day wash out period  - 10 hr fast  - Avoid alcohol and fibre-rich foods  - Consumption of food or drink outside of test not permitted  - Test meal to be consumed within ≤ 15 mins | **Food:**  Yeast-fermented Refined Wheat Crispbread **(Control)** vs. Unfermented Rye Cripsbread (uRCB), Sourdough-fermented Rye Crispbread (sfRCB)  **Amount:**  C: 52g, 880kJ, 6.5g P, 35.0g CHO, 4.1g F  uRCB: 59g, 830kJ, 6.0g P, 35.4g CHO, 0.9g F  sfRCB: 59g, 821kJ, 5.1g P, 36.2g CHO, 1.1g F  **Fibre dose:**  C: 2.9g TF  uRCB: 11.7g TF  sfRCB: 9.5g TF | n = 24 (incl. in analysis)  Hunger and DTE were sig. lower based on AUC measurements post consumption of sfRBC compared to WCB (p < 0.05). | - Postprandial glucose  - Insulin  - GLP-1 | - | 0 | NIL |

**Abbreviations:** A, amilo; AG, arabino-galactan; AX, arabinoxylan; AXOS, arabinoxylan oligosaccharides; AUC, area under the curve; BAR, barley; BC, barley cereal; βG, β-glucan; BA, β-glucan enriched barley; BGL, blood glucose level; BMI, body mass index; BB, bran bread; BF, breakfast; BSC, brown sorghum cereal; CE, cellulose; CE + RS, cellulose and resistant starch; CWB, coarse white bread; CB, corn bran; Cf, cornflakes; CW, cracked wheat; DTE, desire to eat; DF, dietary fibre; ERB, endosperm rye bread; EI, energy intake; E, evolo; FMP, finger millet porridge; FB, Finnish bread; FFA, free fatty acids; FRU, fructan; FOS, fructooligosaccharides; GI, gastrointestinal; GIT, gastrointestinal tract; GB, German bread; GLP-1, glucagon-like-peptide-1; GIP, glucose-dependent insulinotropic polypeptide; GL, glycaemic load; HAW, high-amylose wheat; HDL, high density lipoprotein; HF, high fibre; HFG, high fibre + glucose; HBGO; High β-glucan; HBGX, High β-glucan + extracted β-glucan cereal; HAW-R, High-amylose wheat refined; HAW-W, High-amylose wheat wholemeal; HDP, hydroxypropyl distarch phosphate; iAUC, incremental area under the curve; IF, insoluble fibre; IO, instant oatmeal; II, insulinemic index; ICAM-1, Intercellular Adhesion Molecule; IFB, intermediate fraction bread; IRFB, intermediate rye fraction bread; K, Kaskelott; kJ, kilojoule; LAW, low-amylose wheat; LDL, low density lipoprotein; LFG, low fibre + glucose; LBG, low β-glucan; LAW-W, low-amylose wheat wholemeal; L, lunch; MetS, metabolic syndrome; MBG, Mid β-glucan; MW, molecular weight; OB, oat bran; OF, oat fibre; OC, oatmeal cereal; SO, old fashioned oatmeal; PP, pancreatic polypeptide; PMP, pearl millet porridge; PYY, peptide YY; Pi, Picasso; PL, placebo; PD, polydextrose; PFC, prospective food consumption; PR, Pumpernickel rye; RDS, rapidly digestible starch; RTEC, ready to eat cereal; RC-Coarse, reconstituted whole grain wheat flour with coarse bran; RC-Flour, reconstituted whole grain wheat flour with fine bran; RSC, red sorghum cereal; RG-Flour, refined grain flour; RRC, refined rice cereal; RRM, refined rice snack mix; RWB, refined wheat bread; RWP, refined wheat pasta; RS, resistant starch; RS+P, resistant starch + pullulan; RWS, resistant wheat starch; RMR, resting metabolic rate; RBB, rye bran bread; RB, rye bread; RK, rye kernels; RP, rye porridge; SOP, Scottish oats porridge; SE, semolina; SCFA, short chain fatty acids; SRFB, sifted rye fraction bread; SCF, soluble corn fibre; SCF+P, soluble corn fibre + pullulan; SF, soluble fibre; sfRCB, sourdough fermented rye crispbread; SWF, standard wheat flour; SBC, Sustagrain barley cereal; SBM, Sustagrain barley snack mix; FT5, Tea with 5g Fibersol-2; FT10, Tea with 10g Fibersol-2; TSH, thyroid stimulating hormone; TC, total cholesterol; TF, total fibre; TGs, triglycerides; uRCB, unfermented rye crispbread; VAS, visual analogue scale; V, vicello; WMS, waxy maize starch; WB, wheat bread; WC, wheat cereal; WCB, wheat crisp bread; WF, wheat flour; WBC, wheat-barley cereal; WR, white rice; WSC, white sorghum cereal; WG, whole grain; WGR, whole grain rye; WGRB; whole grain rye bread; WG-Flour, whole grain wheat flour; WWC, whole wheat cereal; WWF, whole wheat flakes; WWM, whole wheat snack mix; WWB, wholegrain wheat bread; WWP, whole grain wheat pasta; W, wholemeal; WP, wholemeal pasta; WS, wholemeal soda.

*****Interpretation of quality rating: + positive, **ø neutral**
All studies were deemed relevant to the Australian population / context.
All studies were conducted in a laboratory.
